# Application of deep learning in prognostic prediction of non-small cell lung cancer

**DOI:** 10.3389/fcell.2026.1878798

**Published:** 2026-07-17

**Authors:** Xiaoqi Lun

**Affiliations:** School of Airspace Science and Engineering, Shandong University, Weihai, China

**Keywords:** convolutional neural network, deep learning, graph convolutional network, multimodal fusion, multiple instance learning, non-small cell lung cancer, prognosis prediction

## Abstract

Non-small cell lung cancer (NSCLC) is characterized by high heterogeneity. Traditional prognostic evaluation methods such as TNM staging are difficult to accurately characterize the survival differences of patients, and there is an urgent clinical need for more efficient individualized prediction tools. With its powerful automatic feature extraction and nonlinear modeling capabilities, deep learning has shown significant advantages in the processing of multimodal medical data and has become a core technical direction in NSCLC prognosis research. This paper reviews the research progress of deep learning in NSCLC prognosis prediction, sorts out the technical characteristics and challenges of core modalities such as CT/PET-CT imaging, pathological whole-slide images, genomics and clinical data, analyzes the design logic and applicable scenarios of key architectures including 2D/3D convolutional neural networks, multiple instance learning, graph convolutional networks, deep survival models and Transformers, and expounds the evolution path of multimodal fusion from early concatenation and late fusion to attention interaction, tensor fusion and graph interaction modeling. It also introduces the application status of model interpretability techniques such as Grad-CAM and SHAP. On this basis, this paper summarizes the bottlenecks of current research in data heterogeneity, small-sample overfitting, insufficient verification of interpretability, lack of deep multimodal fusion and weak clinical generalization verification, and prospects future development directions. This paper aims to provide technical references for researchers in the field of medical artificial intelligence and facilitate the development of clinically translatable high-precision NSCLC prognosis prediction models.

## 1 Introduction

Lung cancer is one of the leading causes of cancer morbidity and mortality globally and its subtype of non-small-cell lung cancer (NSCLC) accounts for about 85% (Sung et al., 2021). Factors such as tumor stage, histological subtype, gene mutation status, tumor microenvironment, along with the patient’s conditions all exert complex effects on the patient’s prognosis. The prognosis mainly refers to the potential course of the disease and future outcome such as overall or disease-free survival (Pao and Girard, 2011; Travis et al., 2011; Chen et al., 2014). Clinical decision-making is often heavily dependent on conventional tumor-node-metastasis (TNM) staging. And there are frequently significantly differential clinical outcomes for patients at the same tumor stages. Such tumor heterogeneity reflects the shortage of traditional methods in describing this complexity and thus the urgent need to create more accurate and individualized prognostic evaluation methods (Gan et al., 2025; Han et al., 2025).

At present, the widely used technologies such as high-throughput sequencing, digital pathology, and imaging and so on generate extensive clinical data, which is conducive to the establishment of data-driven prognostic models (Weinstein et al., 2013; Aerts et al., 2014). These data include computed tomography (CT) and positron emission tomography/computed tomography (PET-CT) macroscopic images, microscopic whole-slide images (WSI), along with genomic and transcriptomic data at the molecular level. These data possess inherent characteristics of high dimensionality, high noise, obvious nonlinearity, and strong heterogeneity, which brings great difficulties to the prognostic modeling. Traditional statistical models like Cox proportional hazards regression and classic machine learning approaches such as random survival forests are restricted by rigid model assumptions and structural limitations, showing obvious defects in processing such complex data. Cox proportional hazards regression can only accept fixed-dimensional feature vectors as input and is unable to conduct end-to-end analyses on unstructured medical images with variable scales like WSI and 3D CT, nor can it autonomously extract the underlying multilevel spatial structure and semantic information. As far as these high-dimensional genomic data concerned, traditional models primarily employ the strategies of preset feature selection or dimensionality reduction such as least absolute shrinkage and selection operator (LASSO) regularization. These processes can easily destroy the intrinsic co-expression and regulatory networks among genes, thus hard to effectively capture the complicated nonlinear correlations or variable interactions. The derived models then lack the capability to discover the in-depth biological information, restricting the further promotion of prognostic prediction accuracy (Ching et al., 2018a).

**FIGURE 1:**
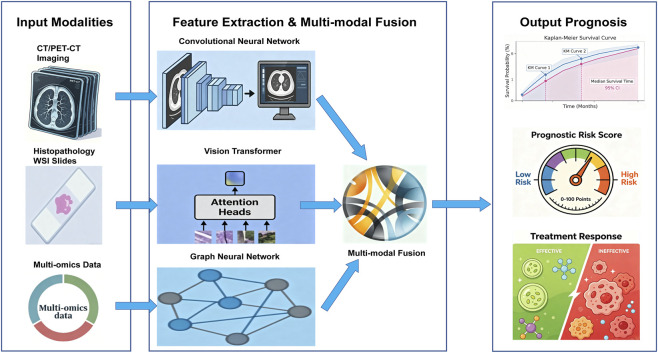
Flowchart of deep learning-based prognostic assessment for NSCLC. The framework consists of three consecutive stages. Left panel (input modalities): the model takes multimodal data as input, including CT/PET-CT imaging, WSI images, and multi-omics molecular profiles. Middle panel (feature extraction and multimodal fusion): CNNs and ViT are used to extract modality-specific features, which are then integrated via attention mechanisms and GNNs to capture cross-modal interactions and spatial topological relationships. Right panel (output prognosis): the fused representation is used to generate three types of prognostic predictions: (i) Kaplan-Meier survival curves with time-to-event analysis, (ii) prognostic risk scores that stratify patients into low-risk and high-risk groups, and (iii) predicted treatment response classified as effective or ineffective.

As a branch of machine learning, deep learning employs neural network architectures with multiple layers of nonlinear modules. These architectures contribute to the automatic learning of hierarchical feature from raw data, which in turn cuts down considerably the need of manually marked features (LeCun et al., 2015). Deep learning has been extensively applied in prognostic prediction of NSCLC and exhibited superior predictive performance compared to conventional ones (Esteva et al., 2019; Boehm et al., 2022). To predict the survival risk, recurrence probability, and response to specific therapies of patients (e.g., immunotherapy), researchers have developed numerous models (Chai et al., 2025; Rakaee et al., 2025) based on CT images (Hosny et al., 2018; Mukherjee et al., 2020), WSI (Campanella et al., 2019; Yao et al., 2019; Li et al., 2025), genomic data (Ching et al., 2018b; Katzman et al., 2018), or a combination of them (Chen et al., 2022; Vanguri et al., 2022). Figure 1 presents a typical deep learning workflow for the prognostic assessment of NSCLC. And Table 1 summarizes technical comparisons of current prevailing deep learning architectures applied to NSCLC prognosis prediction.

**TABLE 1:** Technical comparison of different deep learning architectures in prognostic prediction of NSCLC. For each architecture, the table summarizes the applicable data modalities, core technical mechanisms, key advantages, main limitations, representative studies, Sample size, performance metrics, preprocessing differences, and validation procedures.

Architecture category	Applicable data modalities	Core technical mechanism	Key advantages	Key limitations	Representative literature	Sample size	Performance metrics	Preprocessing differences	Validation procedures
2D CNNs	CT images (multi-planar reconstruction), pathological images	Extract 2D local spatial features via convolution kernels, gradually down sample through pooling layers, and learn translation-invariant features	Mature architecture, high computational efficiency, abundant pre-trained models (ImageNet), suitable for processing single-layer 2D slice data	Cannot directly utilize 3D spatial context information; 3D data need to be dimensionally reduced, which may lose tumor invasion features along the Z-axis	Mukherjee et al. (2020)	129/185/311/84 (four independent cohorts)	C-index: 0.62/0.62/0.62/0.58 (across four cohorts)	3D volume of interest was extracted centered on the largest lung lesion at 64 × 64 × 64, and manually annotated nodule masks were used as input. During training, data augmentation included random cropping, random left-right/up-down flips, and random brightness shifts (0.5–1.0).	First, round-robin training and testing on three independent cohorts (training on two, testing on the third); then, training on the combined three cohorts and validating on a fourth external cohort. In each training run, 20% of samples served as a validation set, and the process was repeated 20 times to select the model with the lowest validation loss. Survival prediction was evaluated using concordance index, Kaplan-Meier curves, and log-rank tests. For malignancy classification, ten-fold cross-validation was used to assess transfer learning performance.
3D CNNs	CT, PET/CT 3D volume data	3D convolution kernels extract spatial and depth-dimensional features simultaneously; residual connections alleviate gradient vanishing; transfer learning mitigates small-sample issues	Fully preserves 3D spatial structural information of tumors; captures 3D spatial interactions between tumor, blood vessels, and pleura	High computational and storage overhead; tumors usually need to be cropped to fixed size, potentially losing partial edge fine details	Tas and Yavuz (2024)	373	AUC = 0.7768	Extracting GTV-1 tumor regions, converting tumor boundary coordinates from mm to pixels, and normalizing images to JPEG format; segmentation errors (mislabeling, interpolation errors between slices, and multiple tumors per slice) handling; grayscale input (240 × 240) constructed and normalized to [0,1].	Dataset split 85%/15% for training/testing with class balance maintained, 5-fold cross-validation. Training uses SGD, up to 200 epochs, with learning rate decay and early stopping. ROC-AUC as evaluation metrics. Ablation studies were performed.
GCNs	Pathological WSI, genomic networks	Construct pathological patches/cell nuclei as graph nodes, update node features via neighborhood aggregation and message passing, capture spatial topology and local community structure	Explicitly models spatial arrangement of cells and topology of tumor microenvironment; captures spatial interactions ignored by MIL; heterogeneous graphs can integrate multiple cell types	Lack of unified standards for graph construction strategies; high overhead for large-scale graph computing; sensitive to hyperparameters (neighborhood definition, graph size)	Zhao et al. (2023)	TCGA- LUAD WSI: 522; Shanghai Chest Hospital NSCLC (SCH-NSCLC) WSI: 696	TCGA-LUAD C-index:0.650 ± 0.027; SCH-NSCLC C-index: 0.642 ± 0.025	First extracting tissue masks from WSI and removing redundant background areas. The foreground is then divided into non-overlapping patches at 512 × 512 × 3. A pretrained ResNet50 used as a feature extractor: the model is truncated after the third residual block, and global average pooling is applied to encode each patch into a 1024-dimensional feature vector.	Employing a five-fold cross-validation strategy at the patient level. One-fold used as the independent test set, while the remaining four folds for training. From the training set, 20% of the samples are randomly selected as a validation set for hyperparameter tuning. Early stopping is applied when the concordance index (C-index) on the validation set ceases to improve.
ViT	CT images, pathological WSI	Self-attention mechanism captures global long-range dependencies in images; positional encoding preserves spatial information; supports variable-size input	Breaks the receptive field limitation of CNNs, captures global context; multi-head attention focuses on different regions; Transformer-XL captures long-range associations	Self-attention computation grows quadratically with sequence length; relies on large-scale pre-training; challenges in interpretability	Ali et al. (2023)	Not applicable	Not applicable	Not applicable	Not applicable
Deep cox network	Genomic/transcriptomic data, clinical structured data	Fit Cox proportional hazards model with neural networks, optimize partial likelihood loss; fully connected layers model nonlinear relationships, output log hazard ratios	End-to-end learning, directly handles right-censored survival data, output interpretable as hazard ratios in traditional Cox models	Relies on proportional hazards assumption; prone to overfitting under small samples; limited interpretability due to lack of biological prior constraints	Wang et al. (2026)	1,012 (discovery) and 361 (validation)	Discovery cohort, C-index = 0.62; external validation cohort, C-index = 0.60	Using univariate Cox regression to identify features significantly associated with OS (P < 0.05) or of clinical importance. Multivariable Cox forward stepwise regression was then applied to select nine independent prognostic factors. The discovery cohort (MSK-ACCESS, n = 1,012) was randomly split into training and testing sets at a 7:3 ratio. ctDNA positivity was defined as at least one non-synonymous somatic mutation at VAF ≥0.1%.	The model was validated using both internal and external strategies. Internal validation was performed on the testing set of the discovery cohort to assess discrimination and calibration via time-dependent AUC and the C-index. External validation used a completely independent ctDx Lung cohort, which was not involved in any training or hyperparameter tuning. The model’s generalizability and robustness were confirmed by achieving 12-, 18-, and 24-month AUCs of 0.67, 0.73, and 0.72, respectively, along with a significant OS difference between high- and low-risk groups (HR = 0.42, P < 0.001).
Transformer (Genomic)	Genomic/transcriptomic data	Treat gene expression as a “sequence”, capture global correlations between genes via self-attention; masking mechanism handles missing values; supports large-scale self-supervised pre-training	Natively handles high-dimensional sparse data; captures long-range gene interactions; pre-trained models (e.g., Geneformer) improve small-sample generalization	High computational cost when sequence length exceeds tens of thousands; interpretation of “attention weights” requires caution; large model size prone to overfitting	Theodoris et al. (2023)	N = 1,063 (human cell-type regulatory networks; not NSCLC survival)	Not applicable (methodology paper on gene network prediction, not NSCLC prognosis)	Gene expression normalization; pre-training on massive single-cell data; fine-tuning on network prediction tasks	Cross-validation within gene expression compendia (no clinical survival validation)

**FIGURE 2:**
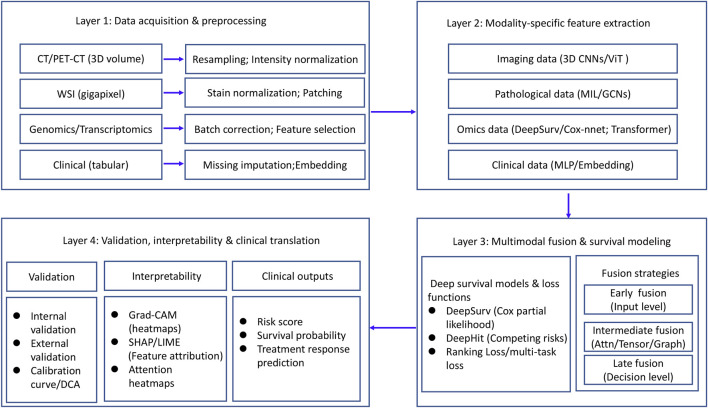
A comprehensive workflow for deep learning-based prognosis prediction in NSCLC. The workflow is divided into four consecutive layers. Layer 1 (data acquisition and preprocessing) covers four main data types and their respective preprocessing steps: spatial resampling followed by intensity normalization for CT/PET-CT; stain normalization followed by patch cropping for WSI; batch effect correction and feature selection for omics data; and missing value imputation followed by categorical variable embedding for clinical data. Layer 2 introduces feature extraction methods organized by data modality: 3D CNNs and ViT for imaging data; MIL and GCNs for WSI; DeepSurv, Cox-net, and Transformer for genomic/transcriptomic data; and MLP for structured clinical data. Layer 3 illustrates three fusion strategies, followed by deep survival models and their corresponding loss functions. Layer 4 (validation, interpretability, and clinical translation) includes model validation (internal cross-validation, multicenter external validation, calibration curves, and DCA), interpretability techniques (Grad-CAM can be used to generate visualization heatmaps for CT and pathology images, SHAP and LIME for feature attribution analysis of genomic and clinical data, and attention heatmaps for localizing lesions in WSI), and final clinical outputs (risk score, survival probability, and treatment response prediction).

We identified relevant literature through targeted searches within PubMed, Web of Science, and Google Scholar using keywords like “deep learning,” “non-small cell lung cancer,” “NSCLC,” “prognosis prediction,” “multimodal fusion,” and “survival analysis.” We prioritized peer-reviewed articles that proposed novel network architectures, achieved notable performance improvements, or addressed critical technical bottlenecks in NSCLC prognostic modeling and that were published in high-impact journals and conference proceedings, for inclusion in this review. Here, “high-impact journals” are defined as those ranked in the first or second quartile (Q1 or Q2) in relevant subject categories according to Journal Citation Reports (JCR). “High-impact conference proceedings” refer to internationally recognized top conferences focusing on medical image analysis, computational pathology and artificial intelligence, including Medical Image Computing and Computer-Assisted Intervention (MICCAI), International Symposium on Biomedical Imaging (ISBI), Conference on Computer Vision and Pattern Recognition (CVPR), International Conference on Computer Vision (ICCV), European Conference on Computer Vision (ECCV), Association for the Advancement of Artificial Intelligence Conference (AAAI), and Conference on Neural Information Processing Systems (NeurIPS), etc. This paper reviews, compares, and analyzes existing research from four core technical dimensions: data modality, unimodal model architecture, multimodal fusion strategy, and model interpretability. It focuses on summarizing and analyzing core network modules including 2D/3D convolutional neural networks (CNNs) (Hosny et al., 2018; Yao et al., 2019; Mukherjee et al., 2020), graph convolutional networks (GCNs) (Li et al., 2018; Zhao et al., 2023), and attention mechanisms (Ilse et al., 2018), as well as modeling and learning strategies such as multiple instance learning (MIL) (Couture et al., 2018; Campanella et al., 2019) deep survival networks (Ching et al., 2018b; Katzman et al., 2018), and multi-task learning (Christ et al., 2017; Shao et al., 2024). Through the above analysis, this paper aims to reveal the applicable scenarios, advantages and limitations of different technologies, provide a technical roadmap for subsequent research in this field, and prospect future development directions. Figure 2 presents the entire workflow, from data acquisition and preprocessing, through feature extraction and multimodal fusion, to survival modeling, validation, and clinical translation.

## 2 Data modalities and technical challenges in prognostic prediction of NSCLC

The foundation of any deep learning model is the data used for its training. In prognostic research of NSCLC, the following major data modalities exist, each with unique dimensions, information content and processing challenges. An in-depth understanding of these modalities is a prerequisite for designing an effective deep learning pipeline.

### 2.1 Clinical and demographic data

Prognostic models mainly depend on clinical and demographic data which are usually presented in structured tables. The key components of such data usually include patient demographic information, lifestyle factors, disease characteristics, and treatment information (Goldstraw et al., 2016; Detterbeck et al., 2017), often combined with continuous and categorical variables. Traditional Cox regression models are capable of coping with such data, while hard to capture the underlying nonlinear relationships and high-order interactions. By contrast, deep learning models employ embedding layers to transform categorical variables into dense vectors and preserve the intrinsic associations among features. This ensures its effective adaption to nonlinear patterns through fully connected layers. For the longitudinal clinical data such as time-varying biochemical indicators, recurrent neural networks (RNNs) or Transformer architectures are more suitable for time-dependent features capture to promote the accuracy of prognostic predictions (Richman et al., 2024). However, the completeness and standardization of these data still restrict the application of this approach. Clinical data usually contain random or non-random missing values and various institutions have their own requirements for data collection respectively. These problems hinder the models’ capacity of generalization greatly.

### 2.2 Genome and transcriptome data

Molecular profiling is a key source of tumor precise prognosis. Genomic data, mainly derived from next-generation sequencing, has the characteristics of relatively static genetic alterations such as somatic mutations and copy number ones. Such data tends to be high-dimensional since only a few genes harbor mutations within the thousands of genes detected. Mutation statuses of key driver genes like EGFR, KRAS, and TP53 have been validated as important biomarkers for prognosis (Cancer Genome Atlas Research, 2014; Reck et al., 2016; Hirsch et al., 2017). Transcriptome data, obtained from RNA sequencing or microarrays, can provide a “snapshot” of the cellular functional status at a certain time point and reflect the activities of various biological pathways. These extremely high-dimensional and continuous data possess high information density but also high noise (Simon et al., 2003). In the context of high-throughput technologies such as RNA sequencing, batch effects, biological heterogeneity, and technical noise are intertwined, posing significant challenges to the stability of high-dimensional survival analysis models (Herrmann et al., 2021).

The core technical challenges of such omics data are the curse of dimensionality and the small-sample high-feature problem. Clinical NSCLC sample sizes are usually only several hundred cases, whereas gene expression features can reach tens of thousands of dimensions, exhibiting a typical N≪P distribution, which readily leads to model overfitting (Ching et al., 2018b). From the perspective of computational learning, NSCLC prognosis prediction can be rigorously formalized as a right-censored survival analysis problem under high-dimensional and small-sample conditions. Define the gene expression matrix as X∈R^N×P^, where N denotes the number of patient samples and P denotes the number of gene features; rows of the matrix correspond to individual patients and columns correspond to the expression level of a single gene. Each sample is matched with a censored survival label (Ti, δi), and Ti represents the follow-up observation time, with δi ranging from 0 to 1 indicating whether an event occurs (Travis et al., 2011). This model is primarily to estimate the conditional hazard function via learning a mapping function. The main challenges include establishing a stable model using data with high-dimensional, limited sample size, extensive noise, and feature redundancy, as well as capturing the key biological signals closely related to tumor prognosis while reducing overfitting. This will provide theoretical support for the construction of deep survival models like DeepSurv and Cox-nnet.

### 2.3 Medical imaging data

Medical imaging is foundation for cancer diagnosis, staging, and treatment response assessment. Deep learning has shown remarkable potential in extracting quantitative information from these images. CT imaging can provide high-resolution information such as tumor morphology, size, edges, internal structure, as well as its relations to surrounding tissues. Its 3D nature means that it must be balanced between computational efficiency and the integrity of spatial information during data processing (Song et al., 2021). How to effectively utilize the 3D spatial contextual information of tumors plays an important role in designing relevant prognostic models (Hosny et al., 2018; Lee B. et al., 2020). PET/CT imaging combines both the anatomical information of CT and the functional metabolic information of PET. Apart from the traditional semi-quantitative indicators, deep learning models can also comprehensively explore the metabolic heterogeneity information within PET images. However, partial volume effect may cause the radioactive counts of small lesions in PET images diluted by the surrounding low metabolic tissues. This results in systematic underestimation and quantitative bias in quantitative indicators such as SUVs. Deep learning method also faces the challenge of physical correction while exploring the heterogeneity. Precise registration of multimodal images and effective fusion of heterogeneous information present significant challenges (Yin et al., 2021; Zhong et al., 2023).

Hematoxylin and eosin-stained WSI of pathology, serving as the gold standard for pathological diagnosis, contains rich multi-level information ranging from nuclear morphology, cellular arrangement pattern, tissue growth pattern to tumor microenvironment (e.g., immune cell infiltration) (Veta et al., 2015; Ning et al., 2025). The core challenge lies in its extremely large image scale (gigapixel level), which makes it impossible to be directly fed into standard CNNs for end-to-end training. Meanwhile, fine pixel-level annotation of WSI is time-consuming and labor-intensive, and can hardly be achieved at scale in clinical practice, rendering the fully supervised learning paradigm relying on strong annotations difficult for widespread application. Against this background, weakly supervised learning, unsupervised learning and self-supervised learning have become key research directions in computational pathology. The three form a complementary and collaborative technical system to jointly address core problems such as scarce annotations, extreme scale and strong heterogeneity in pathological images (Campanella et al., 2019; Chen et al., 2021).

### 2.4 Other emerging modalities

In addition to the core modalities mentioned above, other data types have been gradually integrated into prognostic models.

#### 2.4.1 Electronic health records (EHR)

EHR contains a large amount of unstructured clinical text (such as imaging reports and medical progress notes), which harbors rich information not captured by structured fields. Using natural language processing techniques, especially Transformer-based pre-trained language models (e.g., BERT) and state-of-the-art large language model embedding technologies, key clinical phenotypic information can be extracted from these complex texts. Such in-depth mining not only compensates for the deficiencies of structured data but also provides fine-grained semantic features for prognostic prediction (Lundberg et al., 2018; Lee J. et al., 2020).

#### 2.4.2 Blood biomarkers

Circulating tumor DNA (ctDNA), as a minimally invasive “liquid biopsy” approach, has emerged as a highly promising dynamic prognostic biomarker owing to its unique advantages in detecting minimal residual disease and real-time monitoring of therapeutic response (Assaf et al., 2023). In addition, pre-treatment routine blood indicators (such as the neutrophil-to-lymphocyte ratio) and specific protein biomarkers (such as CEA) are often combined with other modal data to jointly construct multimodal prediction models (Sang et al., 2025).

## 3 Essential architectures and key technologies in single-modal deep learning models

**FIGURE 3:**
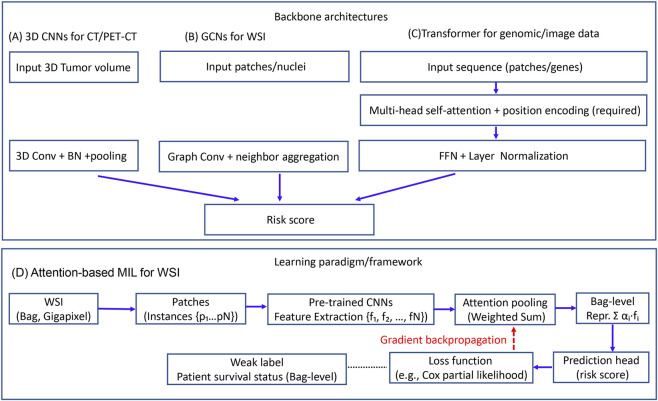
Schematic of representative deep learning methods for NSCLC prognosis prediction. The figure conceptually distinguishes backbone network architectures **(A–C)** from learning paradigms/frameworks **(D)**. **(A)** 3D CNNs: process cropped 3D tumor volumes from CT or PET-CT through successive 3D convolution, batch normalization (BN), and pooling layers to preserve volumetric spatial information. **(B)** GCNs: construct pathology patches or segmented cell nuclei as graph nodes, with edges defined by spatial proximity, feature similarity, or both. Through graph convolution with neighborhood aggregation and message passing, GCNs explicitly model spatial topology and cell–cell interactions within the tumor microenvironment. **(C)** Transformer: treat image patches or gene expression profiles as sequences and employ multi-head self-attention to capture global long-range dependencies. Since the self-attention mechanism is permutation-invariant, positional encoding is required to preserve spatial or sequential information. This architecture overcomes the limited receptive fields of CNNs. **(D)** MIL: a weakly supervised learning framework for gigapixel WSI analysis. The WSI is treated as a “bag” and partitioned into patches serving as “instances.” A pretrained CNN extracts instance-level feature vectors {f_1_, f_2_, …, fN}. These features are aggregated by a learnable attention pooling layer, which computes a weighted sum using instance-specific attention weights {α_1_, α_2_, …, αN} to produce a bag-level representation ∑α_i_·f_i_. This representation is fed into a prediction head to generate a risk score. During training, the risk score is evaluated against bag-level weak labels via a survival loss function, and gradients are backpropagated through the entire framework. Only bag-level labels are required; no patch-level annotations are needed.

For the diverse data modalities mentioned above, researchers have proposed a wide range of deep learning methods. Conceptually, these methods fall into two broad categories: backbone network architectures (such as 3D CNNs, GCNs, and Transformers) and learning paradigms or frameworks, with one typical example being MIL. MIL combines a CNN backbone with an attention aggregator. Figure 3 exhibits the core mechanism behind each approach.

### 3.1 Medical image modeling approaches

#### 3.1.1 Radiomic feature modeling based on 3D CNNs

To fully exploit the three-dimensional spatial structural information contained in CT and PET images, customized backbone network architectures represented by 3D CNNs have become the mainstream technical solution for prognostic prediction tasks of such modalities. Hosny et al. constructed a prognostic prediction model based on three-dimensional residual networks (3D-ResNet), which takes the three-dimensional tumor cube data segmented from pre-treatment CT images of NSCLC patients as input. The backbone network is stacked by multiple residual modules, each containing three-dimensional convolution, batch normalization, and rectified linear unit activation functions. Skip connections can effectively alleviate the gradient vanishing issue during deep network training, and generate risk scores via fully connected layers to estimate patient overall survival (OS) (Hosny et al., 2018). Since medical imaging datasets frequently suffer from insufficient sample volumes, this work therefore adopts a video-driven transfer learning scheme. The 3D network is pretrained on a large natural video dataset, followed by fine-tuning on the target lung cancer CT cohort. Experimental observations demonstrate that such a strategy noticeably strengthens model generalization under limited sample conditions (Hosny et al., 2018).

Similarly, Lee B. et al. (2020) developed a 3D CNN model employing preoperative CT imaging for lung adenocarcinoma (LUAD) recurrence-free survival prediction, and verified the derived risk score as a robust prognostic marker independent of clinical T stage. A central merit of 3D CNN is its capability to reliably characterize the global three-dimensional morphology and invasive features of tumors. However, it has limitations such as high demand for computing resources and the common requirement of cropping or resampling tumor regions to a fixed size, which may lead to the loss of some fine spatial information. To balance predictive performance and computational cost, some studies have adopted a 2.5D strategy, which takes consecutive multi-slice two-dimensional images as input to approximately represent three-dimensional contextual information. This strategy has been widely used in tasks including tumor segmentation and prognostic prediction (Xie et al., 2019). In response to the information loss caused by the fixed input size of 3D CNNs, network architectures based on global average pooling or attention mechanisms developed in recent years can alleviate this limitation to a certain extent and further improve the utilization efficiency of three-dimensional spatial information. On this basis, multi-resolution networks and tumor habitat analysis strategies enable the collaborative representation of multi-scale features. While taking into account cellular-level details and macroscopic structures, they quantify the spatial interactions of intratumoral heterogeneous regions. The integration of such strategies with deep learning has been applied to the accurate prediction of immunotherapeutic efficacy (Caii et al., 2024).

#### 3.1.2 Weakly supervised modeling of pathological WSI images based on MIL

Due to the enormous size of WSI, it cannot be directly input into a CNN. The MIL framework has become a classic paradigm to address this problem (Campanella et al., 2019). In MIL, a single WSI is regarded as a “bag,” and thousands of small image patches cropped from it are regarded as “instances” within the bag. The model only needs to know the label of the entire bag (e.g., the patient’s survival status) without requiring fine annotation of each instance.

Early MIL methods typically first used a pre-trained CNN as the backbone network to extract features of all image patches, then aggregated the features of all instances into a bag-level feature vector via an aggregation function (e.g., max-pooling, average-pooling), and finally fed the vector into a survival prediction head for risk estimation. Although effective, this approach treats all instances equally and ignores the differential contributions of different regions to prognosis.

The attention-based deep MIL proposed by Ilse et al. (2018) provides a novel solution for this field. This method introduces a learnable attention network as a feature fusion module when aggregating instance features, which can dynamically assign a weight to each instance to highlight key image patches that contribute more to the final prediction. The DeepAttnMISL model proposed by Yao et al. (2019) has successfully applied this approach to prognosis prediction of NSCLC. It can not only predict survival risk, but also generate attention maps that highlight histological regions associated with prognosis, such as tumor cell-dense areas or tumor-stroma interfaces, which improves the interpretability of the model to a certain extent.

From the perspective of inductive bias, different MIL pooling functions essentially correspond to different assumptions about the mechanism of prognosis formation. The maximum pooling is based on the existence assumption that bag labels are determined by at least one strongly positive instance, which makes it applicable in tasks that require detecting specific high-risk structures. However, prognostic risk is often not affected by a single factor, but rather the combined effects of multiple tumor regions. The attention-based pooling method calculates the weighted sum of all instances, and the weight allocation is dynamically adjusted based on the actual contribution of each instance. This soft-pooling scheme can capture the global histological patterns and is more consistent with the continuity characteristics of survival risk. This may be a reasonable explanation for the superior performance of attention-based MIL methods in prognostic prediction tasks compared to traditional ones (Ilse et al., 2018; Yao et al., 2019), which still needs further verification through more rigorous ablation experiments or theoretical analysis, however. Employing deep learning to achieve automatic kernel segmentation and morphological feature extraction can objectively and quantitatively evaluate kernel heterogeneity. The extracted morphological, textural, and spatial distribution indicators are closely associated with the OS rate of patients and can provide stable basis for prognostic stratification (Alsubaie et al., 2021; Abel et al., 2024).

#### 3.1.3 Topological relation modeling based on GCNs

The MIL method regards WSI as a set of independent instances, ignoring the key spatial organizational relationships among instances and failing to reflect the inherent topological structure of the tumor microenvironment. In response to this deficiency, researchers introduced the GCNs model to improve it (Li et al., 2018; Zhao et al., 2023). The model takes image blocks or segmented cell nuclei within WSI as graph nodes, establishes connections between nodes based on spatial proximity and feature similarity, and completes the modeling of the entire WSI graph structure. In the GCNs neighborhood aggregation framework, nodes continuously absorb information from neighboring ones to complete feature iterations, ultimately obtaining feature expressions capable of simultaneously characterizing local cell communities and global tissue morphology. The DeepGraphSurv model constructed by Li et al. is an early application attempt under this idea (Li et al., 2018). Zhao et al. subsequently proposed the CoADS model, a cross attention dual space graph network that models node proximity relationships in both physical and feature spaces, and used cross attention modules to achieve dual branch information fusion, achieving better performance in survival prediction tasks (Zhao et al., 2023). The introduction of spatial topology information can effectively boost the performance of WSI driven prognostic prediction models.

#### 3.1.4 Transformer-based modeling

The application of vision transformer (ViT) has addressed the limitation of CNNs in capturing long-range dependencies in high-resolution medical images (Ali et al., 2023). Representative variants of ViT, represented by Swin Transformers, are increasingly being applied in radiomics and pathomics research (Ali et al., 2023). In the integrated modeling of imaging features and clinical indicators, Lian et al. proposed a prognostic model based on the Transformer and graph neural network architectures, which integrates imaging features with non-imaging clinical data (Lian et al., 2022). Their study showed that this Transformer-Graph model improved the accuracy of survival prediction compared with traditional TNM and ResNet-Graph models (Lian et al., 2022). The self-attention and mutual attention network relies on the attention module to achieve bidirectional cross guidance between CT images and genetic data, optimize feature representation, and can be applied to recurrence prediction of tumors such as NSCLC (Ai et al., 2024). The Pathway Aware Multimodal Transformer (PAMT) constructed by Yan et al. (2026) effectively alleviates the “semantic gap” between pathological images and genetic data, achieving precise alignment and fusion of multimodal information at a fine-grained level. This model not only significantly improves the accuracy of survival prediction on multiple tumor datasets, but also has good interpretability and can intuitively locate key lesion areas, providing reliable artificial intelligence basis for accurate diagnosis and treatment of tumors (Yan et al., 2026).

### 3.2 Modeling for genomic and clinical data

#### 3.2.1 Deep survival analysis networks

Combining neural networks with classical Cox proportional hazards models is one of the commonly used approaches for processing censored survival data. The model can learn scoring indicators proportional to risk in an end-to-end framework by optimizing the Cox partial likelihood loss function.

Cox-nnet was first proposed by Ching et al. (2018b) and is an early representative work in this direction. Based on single hidden layer neural network, this method can be directly inputs high-dimensional gene expression data. As the learned feature, the output of hidden layer is then connected to that of standard Cox regression for prediction. Its predictive performance on multiple TCGA cancer datasets is similar to the traditional methods like LASSO Cox and performs better in some tasks.

As a deep Cox model constructed by Katzman et al. (2018) DeepSurv is more versatile in various scenarios. It employs a multi-layer fully connected network as the main structure and can better explore the complex nonlinear correlations within genomic data. With fully connected network-centered and single output node corresponding to the patient’s logarithmic risk ratio, the training process of DeepSurv aims to minimize negative logarithmic partial likelihood. Beyond prognostic prediction, DeepSurv can also analyze the differences in patient risks corresponding to different treatment strategies, providing feasible suggestions for personalized treatment.

Subsequent studies integrated prior information on gene pathways and further expanded this framework, primarily enhancing the biological relevance and interpretability of models like PAGE Net (Hao et al., 2019). In addition to frameworks based on Cox models, discrete-time survival ones divide continuous survival time into several time intervals and thereby transform the original task into an issue of interval probability prediction. This approach relaxes the assumption of proportional hazards and the standard classification loss can be directly applied for model training, thus reducing the implementation difficulty accordingly. DeepHit and other models further promote the prediction accuracy and generalization ability via directly learning the probability distribution of survival time (Lee et al., 2018). Its good generalization performance has been observed in the application of deep survival models regarding cancer genomics (Yousefi et al., 2017).

#### 3.2.2 Modeling strategies for gene expression data

In order to fully utilize the superiority of CNNs in image recognition, many researchers have attempted to convert 1D gene expression data into 2D pseudo images. Wang et al. suggested a method employing the hierarchical information of gene functions in the Kyoto Encyclopedia of Genes and Genomes (KEGG) pathway database to rearrange the selected vital genes into a 2D matrix. Then the gene expression profiles for each patient were converted into corresponding images (Wang S. et al., 2022). Based on this, they constructed a multimodal CNNs model using these images with patient clinical information as input to predict the 5-year OS rate of lung cancer patients. This method attempts to integrate the existing biological knowledge to enable CNNs to capture the functional associations among genes via visual representation. However, such image transformations may impair the genes’ original regulatory relationships and thus affect both the biological interpretability and cross dataset generalization ability. This is also a common challenge for the current methods of pseudo image conversion.

Since genes interact mutually through complex networks, Hongyoon et al. proposed a deep learning method incorporating gene co-expression networks (Choi and Na, 2018). During the training stage, a gene co-expression network using weighted gene co-expression network analysis was first constructed and then the functional modules significantly correlated with patient survival duration were selected. The extracted representative genes within the key modules will be input into a shallow one-dimensional CNNs to realize prognostic risk stratification. This strategy of integrating prior knowledge on gene networks into model feature construction will be conducive to the model’s interpretability and generalization robustness.

## 4 Multimodal data fusion

Single data modality merely reflects partial tumor biology. Integrating multimodal data covering imaging, pathology, genomics, and clinical practice to construct more comprehensive prognostic models has become a dominant direction in this field (Boehm et al., 2022). Multimodal fusion can be conducted at different levels like data, feature, and decision layers, respectively, accompanied by gradually increased technical complexity.

### 4.1 Evolution of fusion levels: early, late and intermediate fusion

Early fusion strategies usually integrate different modal data directly at the model input stage. For example, handcrafted radiomic features extracted from CT images are directly concatenated with clinical variables into a unified feature vector, which is then input into predictive models such as support vector machines or shallow neural networks (Wang C. et al., 2022). Alternatively, on the premise that PET and CT images have completed spatial registration, they are regarded as different channels and concatenated in the channel dimension as the input of 2D or 3D CNNs (Yin et al., 2021). This type of method is simple and intuitive to implement, but has significant limitations: it is difficult to effectively adapt to the inherent differences of heterogeneous data in scale, distribution and statistical characteristics, and cannot fully model the deep and complex interactions between modalities.

Late fusion is a “divide-and-conquer” strategy. First, an independent predictive model is trained for each data modality (e.g., a 3D CNNs model based on CT and a DeepSurv model based on genetic data), and then the outputs of each unimodal model (e.g., risk scores) are fused at the decision level. Common fusion methods include weighted averaging, voting and others (Yin et al., 2021; Zhao T. et al., 2025). The advantage of this method is its high modularity, robustness to the absence of certain modalities, and independent optimization of each unimodal model. However, its main limitation is that it ignores the interactive information between modalities at the feature level.

Intermediate (hybrid) fusion is one of the most concerned and robust methods at present, which can capture complex interactions between modalities at a deeper level while maintaining modality specificity. Its core idea is to first use an independent “subnetwork” to learn a high-level, abstract feature representation for each modality, then fuse these representations at the intermediate layer of the model, and finally input the fused features into subsequent network layers for final prediction. This method can capture complex interactions between modalities at a deeper level while maintaining modality specificity.

### 4.2 Cutting-edge fusion technologies: attention, tensor and graph interactive modeling

The simplest form of intermediate fusion is to directly concatenate feature vectors output by different sub-networks into a long vector (Mobadersany et al., 2018). The work by Mobadersany et al. is representative of this direction, in which they directly concatenate features extracted from pathological images by CNNs with key genomic features (such as IDH1 mutation status) to predict the survival of glioma patients, and its performance outperforms the corresponding single-modal models.

The attention mechanism can dynamically weigh the importance of different inputs, so it has been extensively studied in multimodal fusion. The dynamic attention and masking model (DyAM) proposed by Vanguri et al. can learn attention weights for each modality separately, and thus adaptively focus on richer data sources from different patients (Vanguri et al., 2022). As a more complex fusion framework, pathological fusion can simultaneously cope with the modality loss problem via integrating pathological images and genomic features (Chen et al., 2022). The key computational challenge of multimodal fusion is how to realize the semantic alignment and deep interaction of heterogeneous data within the feature space. Taking pathological and CT images, for example, there exist differences in scale and semantics between the microscopic cellular morphological features of the former and the macroscopic tumor structural ones of the latter. If we only depend on feature connections, it will be difficult to effectively establish a correspondence between the both. Pathological fusion introduced a common attention mechanism for this (Chen et al., 2022). It can achieve the bidirectional interactions between image features and genomic ones in the public semantic space and dynamically update each other through cross modal attention layers. This will contribute to the alignment of fine-grained cross modal features and more accurate capture of key cross- modal associations. To some extent, this mechanism also simulates the diagnostic process of clinical doctors evaluating tissue morphology based on gene mutation data.

Pathomic Fusion also introduces a tensor-based fusion method to better simulate high-order interactions among modalities (Chen et al., 2022). Specifically speaking, it constructs high-order feature tensors from feature vectors of different modalities through outer product. Since each element in the tensor represents the multiplicative interaction between different modal features, it is possible to capture richer cross modal relationships in comparison with simple connections. Despite the high computational cost and dimensionality explosion issues of this method, it demonstrates great potential in capturing deep semantic interactions.

Some frameworks utilize shared latent feature representations to achieve joint prediction of multiple prognostic outcomes. As reported in a study by Cui et al. (2021) variational autoencoders (VAEs) were used to the map omics data including PET radiomics data, cytokines, and microRNAs to a unified latent space, followed by fusion with dosimetric features extracted by one-dimensional CNNs to achieve joint prediction of radiation-induced pneumonia and local tumor control. This work is a typical instance for employing shared latent representations to align heterogeneous data and support multi task prediction (Cui et al., 2021). The multimodal patient embedding and other representation learning modules can markedly improve the performance of survival analyses through aligning CT, PET, and RNA-seq data into the same feature space (Farooq et al., 2025).

Graph neural networks (GNNs) has been applied by many studies to simulate the complex correlations among modalities. Each modality can be regarded as a node in the graph and the connection edges among modalities are constructed dependent on the prior knowledge or inherent correlation within the data. The enhanced modal representations fused with other modal information are then learned via GCNs and other GNNs. Current multimodal fusions are gradually breaking away from the traditional correlation-dependent paradigm and focusing more on the robustness and universality. Causal inference can effectively differentiate causal driving factors and false correlations among modalities, which greatly promotes the model’s generalization ability and clinical reliability across multiple centers and devices scenarios. Castro et al. systematically verified the effectiveness of causal modeling in improving the robustness of medical image analysis frameworks. They further extended causal representation learning to multimodal medical data fusion, where spurious correlations across modalities were identified and removed using structural causal models (Castro et al., 2020). Through self-supervision pre-training, multimodal foundation models can realize global unity and depth alignment of cross-modal representations during pre-training. The BiomedCLIP model proposed by Zhang S. et al. (2025) achieved semantic alignment among multiple modalities after pre-training on 15 million medical image text pairs, and achieved excellent performance in multiple downstream tasks. The MAIRA model proposed by Srivastav et al. (2024) demonstrates how to adapt the basic models to specific medical tasks such as radiology report generation and achieve efficient deployment in a lightweight manner. These “pre training + lightweight fine-tuning” modes effectively simplify the fusion complexity in downstream tasks and promote the transformation of multimodal medical artificial intelligence towards generality and adaptability (Srivastav et al., 2024).

### 4.3 Missing modality processing and multi-task learning

As is known, patients’ multimodal clinical information is frequently incomplete. Cheerla and Gevaert (2019) proposed a multimodal whole cancer prognosis prediction model via unsupervised representation learning. To mitigate the problem of modal loss, this model introduces a multimodal dropout strategy. Some modal data will be randomly discarded during training to force the model to learn the inference of a unified patient representation vector based on any available subset of modalities. Even in the presence of merely partial modal inputs, stable survival prediction can still be made. Unlike Siamese networks or similarity loss functions, this model relies on multimodal autoencoders to fuse different types of data. Another strategy to cope with such missing data is the introduction of auxiliary reconstruction tasks. The SurvNet model proposed by Wang et al. (2020) synchronously optimizes the missing value imputation, survival classification, and Cox regression in a multi task learning framework. It can effectively process common feature missing in clinical data and end-to-end survival risk in the lack of explicit interpolation.

Multi task learning relies on shared representations to strengthen the model generalization ability. Yang et al. (2021) designed a multi-omics series of deep learning models to jointly predict the response of NSCLC patients to PD-1/PD-L1 immunotherapy, as well as progression free or OS. This type of joint learning mode enables the model to concentrate on common features related to treatment outcomes and long-term survival, thereby promoting cross task generalization effectiveness.

## 5 Loss functions, interpretability and data augmentation

### 5.1 Specialized loss functions design for survival analyses

The pivotal task of lung cancer prognosis is survival analyses. The integration of deep learning and survival analyses mainly relies on loss function design and model architecture. As representatives of deep Cox networks, DeepSurv and Cox-nnet fit complex nonlinear relationships through neural networks, capable of processing censored survival data and compatible with traditional survival analysis frameworks. However, their predictive performance extremely depends on the assumption of proportional hazards. Research has found that Cox models exhibit a certain robustness in the case of small deviations from assumptions, and their predictive performance often exceeds expectations practically (Ching et al., 2018b; Katzman et al., 2018).

The discrete-time survival model splits the continuous time axis into several intervals, thus the original survival prediction problem is correspondingly transformed into that of multi classification or distribution learning. This approach bypasses the restrict of proportional hazards and acquires greater flexibility during training. DeepHit employs a deep survival loss function based on competitive risk, and DeepBTS further differentiates supervised from semi-supervised learning loss function configurations. DeepBTS exhibits more superior performance in disease-free survival predictions (Lee et al., 2018; Lee B. et al., 2020). Another work attempts to promote the accuracy of patient risk ranking and more close alignment with clinical assessment indicators. Most of these methods are non-convex, leading to considerable optimization difficulties. And practically they frequently need to be combined with multi task learning. Achieving feature regularization via a shared representation layer, the model’s generalization ability and robustness can be effectively enhanced in the presence of missing data (Yang et al., 2021).

### 5.2 Model interpretability from visualization to feature attribution

The clinical trust and regulatory approval of deep learning are mainly hindered by its nature of black box. The essence of model interpretability is to clarify the intrinsic rationale behind the model’s specific predictions. And this will be conducive to discovering new biomarkers, validating the rationality of models, and promoting clinical translations.

#### 5.2.1 Interpretation based on feature visualization

Gradient-weighted class activation mapping (Grad-CAM) is one of the commonly used visualization techniques (Selvaraju et al., 2020). By calculating the gradients of feature maps output via the last convolutional layer relative to a specific class’s prediction score of, this method produces a heatmap of identical size to the input image, highlighting image regions crucial for the model’s decision-making process. Grad-CAM is commonly applied to interpret the NSCLC prognosis models utilizing CT and histopathological images. For example, Huang et al. (2022) used Grad-CAM to generate “suspicious regions” on CT images and found that the model mainly focused on the internal regions and margins of tumors, as well as the relationship between tumors and the pleura, which is somewhat consistent with prognostic features considered by clinicians. Rakaee et al. (2025) used Grad-CAM to show the WSI regions attended by their model predicting immunotherapy response, and found that these regions were associated with the density and distribution of tumor-infiltrating lymphocytes.

Nevertheless, visual interpretation methods such as Grad-CAM still possess inherent limitations. The feature map of the last convolutional layer is the main reliance for interpreting the results of this type of method. Its low spatial resolution results in relatively rough heatmaps, which makes it difficult to accurately locate tiny but critical tissue structures like tumor infiltrating lymphocytes. The heatmap often cannot effectively reflect the truly essential input region in the prediction in the case of saturate gradient induced by too high confidence in predicting a certain category. In the case of multiple regions of interest with differential sizes and scattered distributions within an image, Grad-CAM usually only focuses on the region with strongest response and neglects the others with comparable importance. Researchers have introduced a series of enhanced schemes to overcome these shortcomings. The introduction of pixel-level weights by Grad-CAM++ is primarily to resolve the problem of multi-target localization. Score-CAM utilizes forward propagation scores as weighting coefficients instead of relying on gradients, thereby alleviating the influence of gradient saturation. And LayerCAM integrates multi-layer feature maps to produce outputs with higher positioning accuracy.

However, heatmap-based approaches like Grad-CAM only offer *post hoc* visual interpretations of model decisions and cannot reflect the model’s actual reasoning process. Studies have shown that the localization accuracy of Grad-CAM falls considerably short of human experts in the aspect of medical image interpretation. This gap is further magnified especially when dealing with lesions with multiple instances, small volumes, and complex morphologies. Therefore, the heatmaps should not be regarded as the sole decision basis of the model in clinical applications. Several systematic frameworks have been developed for evaluating interpretations methods such as D-RISE and ROAR. These approaches quantify variations in model output before and after input perturbations to validate the reliability of explanations, which provides a more rigorous standard for the evaluations on the authenticity and clinical applicability of heatmap-based interpretability results.

#### 5.2.2 Interpretation based on attention and feature attribution

In attention mechanism-based MIL models (Ilse et al., 2018; Yao et al., 2019) and multimodal fusion models (Chen et al., 2022; Vanguri et al., 2022), the attention weights within the models themselves can provide a certain degree of interpretability. For WSI, the attention weights assigned by the model to each image patch can be mapped back to the original whole-slide image to generate attention heatmaps, which directly indicate which histological regions (such as tumor cell nests, necrotic areas, and stromal regions) are important for prognostic prediction. For multimodal fusion, the attention weights assigned by the model to different modalities including CT, pathology, and genomics reflect which type of information may be more decisive for determining the prognosis of a specific patient.

For tabular clinical or genomic data, feature attribution methods represent one of the primary interpretive approaches. SHapley Additive exPlanations (SHAP) is a game-theoretic feature attribution method based on Shapley values, which calculates a contribution value for each feature of each sample, representing the marginal contribution of that feature to the final predicted value of the sample (Lundberg et al., 2020). SHAP values can be used for global analysis to identify features important to the entire cohort (e.g., ECOG performance status, disease stage), as well as for local interpretation to analyze why a specific patient is predicted to be at high risk (Lundberg et al., 2018; Zheng et al., 2023). Although SHAP values have a solid game-theoretic foundation and ensure additivity and consistency of feature contributions, their computational complexity is high. For high-dimensional genomic data, the exact calculation of the marginal contribution of each feature to all feature subsets is computationally infeasible, and approximate algorithms (such as KernelSHAP or TreeSHAP) are typically employed, which may introduce certain approximation biases in the interpretive results. Different SHAP approximation algorithms handle feature dependence differently. KernelSHAP, a model-agnostic approximate method, usually samples from the marginal distribution, implying the assumption of mutual independence among input features; in contrast, TreeSHAP, a method designed for tree-based models, explicitly models dependencies between features via conditional expectations and is relatively more robust to collinearity. In high-dimensional genomic data, the strong collinearity among features (e.g., gene co-expression networks) may reduce the reliability of SHAP value assignment when KernelSHAP is used. In comparison, TreeSHAP can provide more reliable attribution results when used with tree-based survival analysis methods (e.g., random survival forests). In the SurvivalNet framework developed by Yousefi et al. (2017) a risk backpropagation technique was adopted to backpropagate the model-predicted risk scores to the input layer via gradients, thereby calculating the contribution of each gene feature to the risk score, and successfully identifying known biomarkers associated with prognosis.

### 5.3 Data challenges and mitigation strategies

At present, deep learning in NSCLC prognosis research still faces multiple core challenges, covering data processing, model training, interpretability validation, generalization and promotion, among other dimensions. These challenges are closely interrelated and collectively constitute the main bottleneck for clinical translation.

Data heterogeneity and insufficient standardization are key factors limiting the generalization ability of NSCLC prognosis models. Significant differences exist in imaging acquisition protocols (e.g., slice thickness, reconstruction algorithms), pathological staining schemes, gene sequencing platforms and processing workflows among different medical institutions, which introduce technical batch effects unrelated to biology into the data, thereby impairing the generalization ability of models. Traditional radiomics studies have confirmed the significant impact of multicenter data differences on the performance of prognosis models (Hosny et al., 2018), and this problem is even more prominent in deep learning models (Mukherjee et al., 2020; Howard et al., 2021). For imaging data such as CT, the differences in acquisition protocols can be alleviated by resampling to isotropic voxels, intensity normalization (e.g., Z-score normalization), and standardization of CT value window width and window level. For digital pathological images, staining normalization methods (e.g., StainGAN) can be adopted to convert the staining styles of different centers into a unified reference space. These standardization methods form the basis for mitigating batch effects, yet traditional batch correction methods such as ComBat were originally designed for low-dimensional omics data and have limited applicability to the high-dimensional, non-linear deep features extracted by deep learning. In recent years, domain adaptation techniques have been widely used to address cross-center data distribution discrepancies, which can effectively attenuate the influence of batch effects on the distribution of high-level semantic features through adversarial training strategies. In addition, generative adversarial network-based staining normalization methods (e.g., StainGAN) can convert pathological images from different centers to a unified staining style, helping to reduce feature distribution shifts caused by staining variations. However, the cross-center application of such methods in prognosis models of NSCLC is still relatively limited, and future further research can concentrate on it.

A prominent problem derived from data heterogeneity is the small sample size and overfitting, particularly evident in prognostic studies of NSCLC. Although public databases such as TCGA can provide some data support, there are usually relatively few samples with complete survival follow-up information and high-quality multimodal data in NSCLC related research. The parameters in deep learning models often demonstrate large number, with the mismatch between sample size and model complexity easily leading to overfitting. Especially for rare molecular subtypes of NSCLC, there is a scarcity of high-quality annotated data, not only exacerbating overfitting but also seriously affecting the practical application of the model. At present, transfer learning (Hosny et al., 2018; Mukherjee et al., 2020), data augmentation (Wang et al., 2019), self-supervised learning (Azizi et al., 2023) and model structure simplification (Mukherjee et al., 2020) represent mainstream strategies to address this issue, which can alleviate the limitations caused by small sample sizes to a certain extent.

Insufficient validation of interpretability remains a critical bottleneck hindering the clinical translation of prognostic models for NSCLC. At present, most interpretability methods adopted in existing studies (such as Grad-CAM heatmaps) are mostly *post hoc* explanations. They are not only hard to elucidate the potential causal mechanisms between imaging features and the prognosis of NSCLC, but the authenticity and stability of their explanations have also largely not been rigorously quantitatively validated (Adebayo et al., 2018; Chang L. et al., 2025). This explanation method cannot establish reliable trust in the clinical environment, and there is still room for improvement in the trust of clinical doctors in model decision-making, which to some extent affects the clinical application of the model.

Apart from the aforementioned challenges, multimodal fusion, model generalization, along with inadequate validation processes also greatly hamper the clinical translations of NSCLC prognostic models. Due to largely dependence on feature concatenation and simple interaction, multimodal fusion still lacks unified fusion frameworks and in-depth exploration of semantic associations among modalities. The performance of true multi-scale and multi-dimensional collaborative modeling is still far from adequate because it’s difficult to properly exploit the complementary information of multimodal imaging data and appropriately identify complex biological aspects associated with prognosis. The majority of current NSCLC prognostic studies use retrospective single center approaches. The validation queue and training set still differ significantly in terms of sample size, case composition, and data collection procedures, although some have carried out multicenter external validation. Some studies even have conflicting results. This also indirectly illustrates the necessity of conducting prospective, multicenter, standardized validation in accordance with the Transparent Reporting of a multivariable prediction model for Individual Prognosis Or Diagnosis (TRIPOD) guidelines (van Timmeren et al., 2019). Strictly following the TRIPOD statement (Collins et al., 2015) and the TRIPOD + AI statement for machine learning model optimization (Collins et al., 2024), conducting more standardized prospective or multicenter retrospective validation, covering complete reports on key indicators such as model calibration curves, decision curve analysis (DCA), and cross center generalization errors, serves as an important foundation for smooth clinical application of NSCLC prognostic models.

## 6 Technical comparison and performance evaluation

Distinct deep learning techniques possess advantages and disadvantages in NSCLC prognosis prediction respectively, with significant differences in both applicable scenarios and performance. To provide a comprehensive quantitative overview of the current state of the field, we summarize the representative deep learning studies in Table 2, detailing their data modalities, architectures, sample sizes, clinical endpoints, and reported performance metrics (e.g., concordance index (C-index) and area under receiver operating characteristic curve (AUC)). Although 3D CNNs can fully explore the three-dimensional spatial information of tumors, they have high requirements for image registration accuracy and computational resources (LeCun et al., 2015; Reck et al., 2016), and their performance largely depends on the accuracy of tumor segmentation. Attention-based multi-instance learning can perform pathological image analyses and locate key prognostic regions under weak supervision, while patch sampling strategy, image quality, and staining consistency all exert significant impacts on its performance (Campanella et al., 2019; Zhong et al., 2023). For NSCLC, this technique can predict the risk of recurrence in patients merely through the attention focusing mechanism that provides sliding horizontal labels (Lu et al., 2021). GCNs are good at modeling the spatial topology of pathological images and capturing various interactions in the tumor microenvironment, but the lack of a unified graph construction standard leads to unstable results (Li et al., 2018; Zhao et al., 2023; Sang et al., 2025). Deep Cox networks support end-to-end modeling of omics data, but overfitting issues are prone to occur in small sample scenarios, and interpretability is rather modest (Ching et al., 2018b; Katzman et al., 2018; Hao et al., 2019). The gene expression imaging method utilizes the powerful feature extraction ability of convolutional networks to achieve modeling, but the data rearrangement process may lead to distortion of biological information (Wang S. et al., 2022). Multimodal strategies can adaptively mine correlations among modalities and have some compatibility with missing modalities, with complex model architecture and more difficulties in training, however (Goldstraw et al., 2016; Detterbeck et al., 2017; Chang L. et al., 2025). Interpretable tools such as Grad-CAM and SHAP play an important part in image visualization and feature attribution, respectively. Grad-CAM generates a heatmap by backpropagating gradients from the last convolutional layer, which can visually present the image regions of interest to the model. However, the spatial resolution of the heatmap is limited by the size of the feature map in that layer, making it difficult to accurately distinguish between tumor boundaries and inflammatory regions. The computational complexity of SHAP increases exponentially with the number of features, and even if approximate methods such as Kernel SHAP can be used, the efficiency is still not high (Veta et al., 2015; Mobadersany et al., 2018; Cheerla and Gevaert, 2019; Yang et al., 2021; Yin et al., 2021). From the perspective of technological development trends, this field demonstrates four clear directions as follows: gradually transitioning from single modality to multimodal fusion, and improving the accuracy and robustness of prognosis prediction through more refined fusion strategies; Transitioning from fully supervised learning to weakly supervised and self-supervised learning, reducing reliance on large-scale fine-grained annotated data; From a purely predictive “black box” model to an interpretable model, visualization and attribution methods have become standard to enhance clinical credibility and promote mechanism exploration; From manually designing feature engineering to end-to-end deep representation learning. Early NSCLC prognosis prediction mainly relies on manually extracted radiological or pathological morphological features, while deep learning models learn hierarchical representations directly from raw data through convolution, attention, or graph convolution operations. While eliminating the bias caused by manual feature design, it also puts higher demands on data quality, model complexity, and interpretability.

**TABLE 2:** Quantitative summary of representative deep learning studies for NSCLC prognosis prediction. This table covers data modalities, architectures, sample sizes, clinical endpoints, and reported performance metrics across diverse datasets and prediction tasks.

Study	Data modality	Deep learning architecture	Sample size	Clinical endpoint	Performance metrics
Hosny et al. (2018), PLoS Med.	CT	3D CNNs	1,194 (7 independent datasets)	OS	2-year OS from the start of respective treatment for radiotherapy (AUC = 0.70, p < 0.001) and surgery (AUC = 0.71, p < 0.001)
Mukherjee et al. (2020), Nat. Mach. Intell.	CT	Shallow CNNs (LungNet)	129/185/311/84 (four independent cohorts)	OS	C-index: 0.62, 0.62, 0.62, 0.58
Lee B. et al. (2020), Sci. Rep.	Clinical and pathological data	DeepBTS (binned time survival)	training cohort, n = 1,022; external validation cohort, n = 298	Recurrence-free survival (RFS)	C-index = 0.7306; AUC = 0.7677
Tas and Yavuz (2024), Diagnostics	CT	3D CNNs (ResNet-34)	373	OS	AUC = 0.7768
Kar et al. (2023), Cancer Med.	Clinical data	DeepSurv	268 (Stage-I NSCLC)	RFS	C-index for training set: 0.832; Test set: 0.677
Lu et al. (2023), Heliyon	CT	Hybrid CNNs-RNNs	Internal: 1869; External: 285	OS	Inverse Probability of Censored Weights (IPCW) C-index 0.75 (internal), 0.69 (external)
Li et al. (2018), MICCAI	Pathology (WSI)	Graph CNNs (DeepGraphSurv)	TCGA, lung squamous cell carcinoma (LUSC): patient:463, WSI: 535; National Lung Screening Trials (NLST), patient: 263, WSI:425	OS	LUSC: C-index = 0.6606; NLST: C-index = 0.7066
Zhao et al. (2023), Comput. Programs Biomed.	Pathology (WSI)	GCNs + cross-attention (CoADS)	TCGA-LUAD WSI: 522; Shanghai Chest Hospital NSCLC (SCH-NSCLC) WSI: 696	OS	TCGA-LUAD C-index:0.650 ± 0.027; SCH-NSCLC C-index: 0.642 ± 0.025
Wang S. et al. (2022), Front. Genet.	Gene expression data from TCGA, Clinical data, KEGG BRITE, KEGG Pathway	CNNs (pseudo image) + MLP	TCGA: 471; GSE37745: 195	OS	AUC = 0.87
Ching et al. (2018b), PLOS Comput. Biol.	TCGA datasets	Cox-nnet (1 hidden layer)	5,031 patient samples for 10 kinds of cancers	OS	Based on the C-inverse probability of censoring weighted (IPCW) score, Cox-nnet has better overall rankings than other methods (CoxBoost, Cox-PH and RF-S)
Vanguri et al. (2022), Nat. Cancer	CT + pathology + genomics + clinical	Dynamic deep attention-based multiple-instance learning model with masking (DyAM)	247 NSCLC patients	PFS/response	AUC = 0.80
Cheerla and Gevaert (2019), Bioinformatics	Clinical data, mRNA expression data, microRNA expression data and WSI	Deep unsupervised representation learning	20,000+ (TCGA, 20 cancer types)	OS	C-index = 0.78 (pan-cancer)
Yan et al. (2026), IEEE TPAMI	Pathology + genomics	Pathway-Aware Multimodal Transformer (PAMT). (Transformer + pathway)	Not reported for NSCLC-specific	OS	TCGA-LUAD C-index: 0.719 ± 0.044; TCGA-LUSC C-index: 0.704 ± 0.015
Wang C. et al. (2022), Front. Immunol.	CT + clinical	Multi-source feature fusion	1135 NSCLC patients	PD-L1 expression and OS	C-index = 0.89
Wang et al. (2020), Front. Oncol.	Clinical (with missing values)	SurvNet (multi-task learning)	1,137 patients with IB-IIA stage NSCLC	OS	C-index = 0.6003
Zhong et al. (2023), Nat. Commun.	PET/CT	Cross-modal DL signature	Internal 1,911, external 345, prospective 999	Occult nodal metastasis	C-index (occult N1 prediction) = 0.958 (validation set), 0.879 (external cohort) and 0.914 (prospective cohort), C-index (occult N2 prediction) = 0.942, 0.875 and 0.919, respectively
Assaf et al. (2023), Nat Med.	ctDNA (liquid biopsy)	Longitudinal model	466 NSCLC patients	OS	C index = 0.66
Zhao T. et al. (2025), Sci. Rep.	Multi-omics + histology	Decision level fusion	TCGA NSCLC: 443 LUAD cases; 359 LUSC cases	OS	C index = 0.67

## 7 Clinical utility assessment: from methodology to clinical practice

The discrimination (e.g., C-index) and calibration of a prediction model can only reflect the model’s intrinsic performance and cannot answer a core question critical to clinical decision-making: whether acting on the model’s predictions can yield a net benefit for patients that is superior to other strategies, and at what risk threshold such action is worthwhile.

### 7.1 DCA

DCA was first proposed by Vickers and Elkin, 2006. It evaluates the clinical utility of a model across a range of risk thresholds using the net benefit index (Piovani et al., 2023). Net benefit is defined as the true positive proportion minus the false positive proportion weighted by the odds of the risk threshold, quantifying the trade-off between detecting true positives and avoiding unnecessary interventions (Steyerberg and Vickers, 2008). DCA compares the candidate model with the two extreme strategies of “treat all” and “treat none,” reflecting the actual trade-off between benefits and harms in clinical decision-making (Piovani et al., 2023).

In NSCLC prognosis research, DCA has been widely adopted. Li et al. constructed a nomogram model based on data from 1,321 NSCLC patients receiving immunotherapy, incorporating 11 independent risk factors; DCA showed that the model achieved higher net benefit than the extreme strategies over a wide range of risk thresholds (Li et al., 2024). Luo et al. (2023) employed the DeepSurv algorithm to analyze routine blood test features, and DCA confirmed that the higher-order feature model had a net clinical benefit advantage in prognostic prediction. In a subsequent machine learning study with 31,873 patients, DCA again demonstrated the model’s net clinical benefit when applied to treatment recommendations (Luo et al., 2026). Piovani et al. (2023) suggested that researchers should first determine whether the model truly outperforms all alternative strategies across a reasonable range of risk thresholds.

### 7.2 Clinical impact curve (CIC)

The CIC is a practical visual tool for understanding how a prediction model would perform across an entire population. It typically shows two curves that change with the risk threshold: a red curve representing the total number of patients the model classifies as high-risk, and a blue dashed curve indicating how many of those high-risk patients actually go on to experience the outcome (the true positives). The separation between these two lines gives a direct sense of the false positive rate at each threshold, which helps clinical decision-makers strike a balance between the burden of intervening and the number of cases they stand to detect. In prognostic research on NSCLC, the CIC is often used alongside DCA. Jin et al. (2025) developed a deep learning model to predict OS in NSCLC patients with leptomeningeal metastasis and assessed its clinical value using DCA together with calibration curves. In another study, Ma et al. (2024) built a nomogram that incorporates a lung cancer inflammatory index and they evaluated its practical utility with both DCA and CIC.

### 7.3 Cost-effectiveness assessment

A systematic review by Bonci et al. (2025) which followed PRISMA guidelines and included 10 eligible studies, states explicitly that there is currently no evaluable evidence on patient-reported outcomes or cost-effectiveness regarding the use of AI in NSCLC management. This lack of evidence is one of the main barriers hindering the clinical translation of AI prognostic models. Nevertheless, potential economic value can still be gleaned from indirect evidence. By enabling more precise patient stratification, AI prognostic models can help cut spending on ineffective treatments while pinpointing patients who are most likely to benefit from intensified therapy. For instance, in certain patients with early-stage (stage II) NSCLC, machine learning-driven survival models have been shown to effectively identify those who could benefit from adjuvant chemotherapy (Luo et al., 2026). A full framework for assessing cost-effectiveness should cover four dimensions: (i) the incremental costs of model development, deployment, and maintenance; (ii) the resulting changes in treatment costs driven by model-informed decisions; (iii) changes in health outcomes, expressed as quality-adjusted life years; and (iv) the overall assessment expressed as an incremental cost-effectiveness ratio, which can then be weighed against a willingness-to-pay threshold.

### 7.4 Practical considerations for clinical workflow integration

To bring prognostic models from research into everyday clinical use, we need to pay attention to three practical dimensions. First, the model has to fit into existing clinical information systems and it should plug into the electronic health record in real time so that predictions show up right at the point of care. AI decision support works best when it’s deeply embedded in the EHR, able to pull in both structured and unstructured data, spot missing information, and offer relevant guideline recommendations on the spot (Hundal et al., 2026). Second, how the results are presented should not add to the physician’s workload but benefiting clinical decisions. An online interactive tool provided by Li et al. (https://icisnsclc.shinyapps.io/DynNomapp/) mentioned earlier is a nice example of clinical integration, it lets doctors instantly get individualized survival predictions and can assist shared decision-making (Li et al., 2024). Third, prognostic models should not be regarded as independent decision-makers but decision supporting tools. Clinical guidelines and institutional protocols should explain clearly how model outputs should be interpreted, what risk thresholds require an intervention, and what override mechanisms are in place when facing the inconsistence between the clinician’s judgment and the model’s prediction.

### 7.5 Current challenges and future directions

A persistent disconnect still remains between model development and clinical validation. Many studies still report only internal validation metrics, with most of them lacking multicenter external validation and prospective designs. Collins et al. published the TRIPOD + AI statement in 2024, which provides updated reporting standards for machine-learning-based clinical prediction models, but adherence continues to lag (Collins et al., 2024). A systematic review by (Bonci et al., 2025) revealed absent cost-effectiveness evidence for AI in the management of NSCLC. This gap underscores the need to develop methodological frameworks for the economic evaluation of AI-driven prediction tools, thereby future health economic assessments can be properly supported. Future studies should integrate formal economic evaluation with clinical endpoint assessment and, when direct trial data are lacking, use decision-analytic models to extrapolate short-term predictions to long-term cost and quality-of-life outcomes. Utility assessment frameworks must evolve in step with the rapid innovations from multimodal fusion strategies and foundation models. Rigorous evaluation of clinical utility through DCA, CIC, economic evaluation, and integrative scientific approaches is essential to ensure that technological progress translate into meaningful improvements on patient care.

## 8 Challenges and future prospects

As summarized in the whole workflow (Figure 2), today’s deep learning models for NSCLC prognosis go through several stages. Even with technical advances at every stage, the whole pipeline still runs into a number of challenges: data heterogeneity at Layer 1, small-sample overfitting at Layer 2, insufficient multimodal integration at Layer 3, and insufficient interpretability verification and clinical generalizability at Layer 4.

### 8.1 Current challenges

At present, deep learning still faces multiple challenges in the prognostic research of NSCLC. First, the problem of multi-center data heterogeneity is prominent. Differences in imaging acquisition protocols, pathological staining schemes, gene sequencing platforms and processing workflows among different institutions introduce significant batch effects into the data. These technical variations irrelevant to biology may affect the generalization ability of the model (LeCun et al., 2015; Hao et al., 2019). To alleviate multi-center data heterogeneity, privacy computing technologies such as federated learning have attracted attention in recent years, which allow models to be trained on local data and share only model parameters rather than raw data, thereby reducing the impact of batch effects to a certain extent. Existing studies have preliminarily applied the federated learning framework to the prognostic analysis of lung cancer (Huang et al., 2025). In addition, generative adversarial networks (GANs) and domain adaptation methods have been explored to map data from different sources to a common feature space, so as to enhance the cross-center generalization ability of the model. Although these technologies have made progress in the general field of medical image processing (Rieke et al., 2020; Liu Q. et al., 2021), empirical studies specifically targeting prognostic prediction of NSCLC are still relatively scarce. Second, affected by differences in imaging acquisition and reconstruction parameters, the extraction results of radiomics and pathomics features are often biased, and establishing a standardized analysis process is the key to improving model robustness (Hu et al., 2023). Third, high-quality annotated data are scarce, especially with limited sample sizes in rare molecular subtypes, making deep models highly prone to overfitting and impairing practical application effects.

In addition, the validation of interpretability remains insufficient. At present, many studies present heatmaps such as Grad-CAM, yet the fidelity of these explanations (whether the explanations truly reflect the model’s decision-making process) and their stability (whether explanations change drastically under minor perturbations to the input) are rarely subjected to rigorous quantitative verification (Rajkomar et al., 2018). In contrast, game theory–based or local surrogate methods such as SHAP and local interpretable model-agnostic explanations (LIME) provide more quantitative assessments of feature importance, and several studies have attempted to apply them to the interpretability analysis of lung cancer prognosis models (Ribeiro et al., 2016; Lundberg and Lee, 2017). Nevertheless, even for these methods, systematic and rigorous quantitative validation of their fidelity and stability is lacking, making it difficult to establish reliable trust in clinical settings. Meanwhile, most interpretability methods belong to *post hoc* explanations, which can hardly reveal the causal mechanisms underlying the associations between features and prognosis; thus, clinicians’ trust in the models still needs to be improved.

Finally, model generalization and validation are inadequate. Many promising models are developed and tested on retrospective datasets from a single institution, lacking rigorous multicenter external validation and even evaluation on independent internal validation sets (Li et al., 2019). Multimodal fusion remains at the level of feature concatenation or simple interaction, without a unified framework or in-depth mining of semantic correlations, leaving a gap from genuine multi-scale collaborative modeling. Promoting clinical translation requires to carry out more thorough prospective or multicenter retrospective validation following reporting requirements for prediction models like TRIPOD.

#### 8.1.1 Methodological biases and reproducibility concerns

Beyond the data-related and validation issues, the literature on deep learning for NSCLC prognosis prediction also carries some underappreciated methodological biases that weaken the validity and generalizability of the reported findings. A more rigorous look at these sources of bias is needed to help set priorities for future research.

##### 8.1.1.1 Dataset imbalance

Dataset imbalance refers to the uneven distribution of sample sizes across different classes (e.g., different histological subtypes, tumor stages, or survival outcome groups) in the training set, which may lead to degradation of generalization ability, distortion of potential performance evaluation metrics, and increased false negative rate of deep learning prognostic prediction models. In the field of NSCLC prognostic prediction, Gilson et al. systematically investigated the impact of phenotypic bias in training data on the generalization ability of deep learning models. Based on pretreatment CT images of 422 NSCLC patients, the study found that overrepresentation of histological subtypes in training data was most significantly associated with decreased generalization, where overrepresentation of adenocarcinoma led to an AUC decrease of 0.320 (95% CI: 0.296–0.344, p < 0.001), and overrepresentation of squamous cell carcinoma caused an AUC decrease of 0.177; additionally, biases in age, T stage, and N stage resulted in AUC decreases of 0.103, 0.170, and 0.120, respectively (Gilson et al., 2021). This indicates that the imbalance in histological subtype distribution is one of the key biases affecting the true performance of NSCLC prognostic models. To address dataset imbalance, improvements can be made synergistically at three levels: data, algorithm, and survival analysis. At the data level, GANs can be used to synthesize minority class samples to balance the training set. This methodological strategy has been validated in medical image classification tasks. Frid-Adar et al. (2018) demonstrated in liver lesion classification that synthetic images generated by GANs can effectively augment the training set and improve CNNs classification performance. Although that study did not focus on class imbalance, its data augmentation strategy provides a feasible methodological reference for prognostic modeling with small-sample medical imaging, such as in NSCLC. At the algorithm level, if prognosis is converted into a high-risk/low-risk binary classification task, Focal loss, which has been successfully used to address extreme imbalance in object detection, can be adopted. Lin et al. (2017) showed that by reducing the loss weight of well-classified majority samples, training can focus more on difficult minority samples, thereby correcting the learning bias without changing data distribution. For survival analysis, Gensheimer and Narasimhan (2019) introduced a discrete-time survival model called Nnet-survival to handle event sparsity and class imbalance caused by right censoring through direct combination with neural networks. It applies a standard discrete-time survival likelihood as the loss function and no explicit weights are assigned to different outcomes—such as death or censoring—to modify the likelihood. Built on Keras/TensorFlow, the model supports flexible loss customization and mini-batch training, making it a convenient framework for researchers to further explore weighted loss functions or sampling strategies that address event sparsity due to right censoring. The synergistic application of these three approaches can systematically enhance the model’s ability to recognize minority outcomes and improve the reliability of prognostic prediction.

##### 8.1.1.2 Label noise

Label noise in NSCLC prognosis prediction comes from several sources. One of the most common form is incomplete labels caused by missing follow-up information. Although the widely used right-censored data format can partially handle incomplete observation data, deep learning models are similarly sensitive to this kind of noise, which can lead to systematic bias in risk estimation. Wissel et al. (2025) conducted a standardized benchmark of multi-omics cancer survival prediction models and revealed how data completeness conditions including missing multimodal data systematically affect survival prediction performance. During multi-omics integration, the absence of certain modality data can significantly change a model’s predictive performance, while statistical models generally outperform deep learning methods when it comes to survival function calibration. Beyond that, differences in follow-up recording standards across institutions, variations in censoring rates, and the non-randomness of censoring mechanisms can all be regarded as label noise at the organizational level. To address this, preliminary strategies include injecting controlled noise during training to make models more robust, and designing loss functions that are less sensitive to label noise, such as employing ranking-based losses or explicitly modeling the censoring distribution. However, dedicated validation of the above strategies in the field of NSCLC prognosis remains very scarce.

##### 8.1.1.3 Selection bias

Selection bias may arise from selective enrollment of specific populations during the study design phase, convenience sampling in retrospective studies, and inherent differences in the composition of patients admitted to different institutions. A systematic review published by Yang et al. (2026), which included 17 studies on prediction models for postoperative recurrence of early-stage NSCLC, found that seven studies were rated as high risk of bias in the model development phase due to inappropriate sample handling methods, and 13 studies were deemed at high risk of bias in the validation phase because of insufficient test set size (<100 cases) or reliance on apparent performance metrics (Yang et al., 2026). Another systematic review focusing on the prediction of EGFR mutation status also revealed the prevalence of selection bias. This review included 59 studies; 31% of the studies had a high risk of bias in at least one domain, most commonly in the index test and patient selection domains. Only 17 studies (29%) reported independent external validation, and the mean AUC of external validation (0.77) was significantly lower than that of internal validation (0.84). This performance drop strongly suggests that the patterns learned by the models from single-center data may contain domain-specific features related to specific acquisition protocols, making it difficult to maintain the original predictive performance in cross-population scenarios (Liu H. X. et al., 2025). Data splitting strategies also cannot be ignored. In a CT-based survival model for kidney cancer, Flannery et al., showed that simply moving from random splitting to covariate-balanced splitting drove the external validation c-index from 0.56 all the way up to 0.74. This kind of cross-cancer methodological evidence is a clear warning: poor splitting choices can introduce severe selection bias (Flannery et al., 2026). Prognostic studies in NSCLC still rarely spell out critical decisions like how the data were split, which directly undermines the credibility and reproducibility of their findings.

##### 8.1.1.4 Reproducibility

The reproducibility risks in deep learning-based prognostic studies for NSCLC should be examined from two aspects. Regarding feature reproducibility. Wang et al. (2024) found that only 31.17% of radiomic features remained stable upon test-retest, whereas deep learning features achieved a reproducibility rate as high as 84.03%. This indicates that deep learning features are more robust to variations in image acquisition conditions and are better suited as inputs for cross-center models. However, roughly 16% of deep learning features still failed to reproduce, a reminder that not all automatically learned features can genuinely withstand acquisition-related fluctuations (Wang et al., 2024). When it comes to the reproducibility of model training, randomness also plays a non-negligible role. Differences in data splitting, weight initialization, and batch order can cause the same method to yield substantially different performance estimates even on the same dataset. Unfortunately, most current studies neither report whether random seeds were fixed nor show the variation in performance across multiple runs, which inevitably weakens the reliability of the conclusions. Following the TRIPOD guidelines, future work should fully disclose the data splitting scheme, the random seeds used, and the confidence intervals of performance. As for federated learning, it provides a viable pathway for verifying multicenter reproducibility under privacy protection by sharing only model parameters without the need to exchange raw data.

##### 8.1.1.5 Model uncertainty estimation

Model uncertainty estimation is about understanding how confident a model really is in its predictions for individual samples, and in clinical decision-making, that kind of confidence measure is essential. Most prognostic prediction models for NSCLC currently give only point estimates like risk scores or survival probabilities, without any accompanying sense of predictive uncertainty. When the model is not actually confident, this can easily mislead clinical judgment. To tackle this problem, Zanitti et al. (2025) proposed the Multimodal Contrastive Variational Autoencoder (MCVAE). Their approach uses modality-specific variational encoders to capture the uncertainty tied to each data source, and it introduces an adaptive gating mechanism in the fusion bottleneck layer. This mechanism dynamically controls how much each modality contributes to the final prediction, which helps the model stay robust even when some modalities are missing (Zanitti et al., 2025). MADSurv, on the other hand, designs an attention mechanism that enables modality-specific expert encoders to simultaneously learn predictive features and their own confidence, and by dynamically focusing on the most reliable modality for each patient, it can output both annual survival probability sequences and a measure of the model’s accuracy in estimating the survival probability (Zhang E. et al., 2025). Currently, most NSCLC prognostic models exhibit a notable gap in uncertainty quantification; future research should incorporate more uncertainty-aware designs and at least report prediction uncertainty intervals rather than merely point estimates. This is not only a technical requirement for enhancing the clinical credibility of models, but also a fundamental prerequisite for meeting the risk-informed demands of clinical decision-making.

#### 8.1.2 Architecture-specific technical recommendations for addressing methodological barriers

##### 8.1.2.1 2D/3D CNNs

2D/3D CNNs are the core architectures for processing CT and PET/CT images. The main challenges they face include feature distribution shifts caused by differences in multi-center acquisition protocols, the tendency of deep networks to overfit under small-sample conditions, and class imbalance between prognostic groups. To address multi-center data heterogeneity, it is recommended to apply Z-score intensity normalization and CT value window width/window level standardization at the model input. Hosny et al. (2018) demonstrated in an NSCLC prognosis study that their in-house 3D CNNs with standardized image preprocessing achieved AUC values above 0.70 for predicting 2-year OS in an external independent validation cohort. This study adopted a cross-treatment-scenario transfer learning strategy within the same disease to mitigate the difficulty of modeling with small samples: the model was first fully trained on CT data from a large cohort of NSCLC radiotherapy patients, and then the final layers were fine-tuned using limited samples from NSCLC surgery patients. The results showed that the predictive performance of this transfer learning scheme was significantly better than that of models trained from scratch with full supervision on the surgery cohort alone (Hosny et al., 2018). Tas and Yavuz (2024) proposed a 3D-CNNs model incorporating the PEN-BCE loss function, which adds a penalty term on top of the standard cross-entropy to impose additional constraints on false positives and false negatives, thereby strengthening the misclassification cost and improving survival prediction accuracy.

##### 8.1.2.2 Attention-based MIL models

MIL has become the mainstream paradigm for prognostic analysis of whole-slide pathological images, and its core challenges lie in: reliance solely on bag-level weak supervision labels without fine-grained instance-level annotations; neglect of spatial neighborhood correlations among local image patches; and highly imbalanced distributions of key histological regions corresponding to different prognostic outcomes. DeepAttnMISL proposed by Yao et al. dynamically assigns a weight to each image patch via a learnable attention aggregation function, achieving effective localization of key prognostic regions using only OS labels (Yao et al., 2020). In terms of instance correlation modeling, the SCMIL framework proposed by Yang et al. (2024) clusters image patches based on morphological features and spatial location information, captures inter-patch contextual interactions through a sparse self-attention mechanism, and retains only interaction computations among task-relevant patches via the learnable SoftFilter module, thereby concentrating attention on neighborhoods rich in prognostic information while reducing computational overhead. To address sample imbalance across prognostic groups, a direction worth exploring is bag-level resampling strategies, i.e., oversampling entire WSI of patients with rare survival outcomes. This approach promises to adjust the distribution balance at the bag level while preserving spatial dependencies among instances; however, its effectiveness and optimal implementation in weakly supervised survival prediction still require further validation.

##### 8.1.2.3 GCNs

GCNs are used to model the spatial topology of cells in pathology images and genomic regulatory networks. Their technical difficulties primarily include the lack of unified standards for graph construction strategies (diverse node definitions, neighborhood thresholds, and edge weight assignment methods), high sensitivity of the model to hyperparameters, and a high risk of overfitting under small-sample conditions. The cross-attention-based dual-space graph convolutional neural network model (CoADS) model proposed by Zhao et al. (2023) addresses these issues by adopting a dual-space graph construction strategy: in physical space, adjacency relationships are established based on the Euclidean distance between pixel patches to preserve the spatial topology of cell arrangements; in latent space, a graph is constructed based on the cosine similarity of feature vectors to capture associations between regions that are morphologically and semantically similar but spatially non-adjacent; and a cross-attention module is employed to enable information interaction between the two graph branches. This approach overcomes the information fragmentation limitation of traditional single-space graph construction. By fusing complementary graph structures from physical and latent spaces, the model is expected to reduce dependence on a single spatial construction setting and may improve robustness to the selection of hyperparameters such as the neighborhood threshold; however, this speculation has not yet been verified through systematic parameter sensitivity analysis in the study by Zhao et al. (2023). To address overfitting in small-sample scenarios, it is necessary to introduce model regularization techniques suitable for graph-structured data. Current practices mostly adopt node-level Dropout strategies, while graph contrastive learning, as an emerging self-supervised paradigm, has also shown potential in mitigating overfitting; its specific effects on lung cancer pathology images deserve in-depth exploration.

##### 8.1.2.4 Deep cox networks

Deep Cox networks are a classic framework for handling high-dimensional genomic data and censored survival times. The main obstacles lie in the proportional hazards assumption often being difficult to satisfy in real clinical data, as well as the small-sample problem of high-dimensional genomic data leading to overfitting and instability in feature selection. To address deviations from the proportional hazards assumption, Gensheimer and Narasimhan (2019) proposed Nnet-survival, which divides continuous survival time into several intervals and transforms the original survival prediction task into an interval probability prediction task. Its loss function uses a discrete-time likelihood rather than the Cox partial likelihood that depends on the proportional hazards assumption, thereby avoiding biases caused by assumption deviations at the architectural level. DeepHit, proposed by Lee et al. (2018), further introduces a competing risks loss function, demonstrating greater flexibility in predicting multiple-event outcomes. Regarding high-dimensional genomic data processing, Cox-nnet proposed by Ching et al. (2018b) directly feeds high-dimensional gene expression data into a single-hidden-layer neural network for feature representation learning. DeepSurv by Katzman et al. (2018) adopts a multi-layer fully connected network, which not only outperforms traditional Cox models in prognostic prediction, but can also provide a basis for individualized treatment recommendations by simulating survival distributions under different treatment options. When the dimensionality of genomic features far exceeds the sample size, using elastic net or pathway-prior-driven feature screening as a preprocessing step is a common strategy to mitigate the curse of dimensionality and overfitting risk; however, its joint optimization with downstream deep Cox networks still requires careful consideration.

##### 8.1.2.5 Transformer model

Transformer demonstrates unique value in genome sequence modeling and capturing long-range dependencies in pathology images owing to the global receptive field of the self-attention mechanism; however, its high quadratic computational complexity and weak generalization ability with small sample sizes are major obstacles. In pathology image modeling, a hierarchical self-attention architecture is recommended, such as the Swin Transformer proposed by Liu Z. et al. (2021), which reduces the computational complexity from quadratic with respect to the full image to linear with respect to the patch size through shifted window operations. Ali et al. (2023) pointed out in their review that Swin Transformer is the mainstream ViT architecture in lung cancer imaging research. Meanwhile, multiple existing studies combine ViT with traditional CNNs and apply them to the imaging diagnosis and prognosis analysis of lung cancers such as NSCLC. In genomic data modeling, the pathway-aware multimodal Transformer (PAMT) proposed by Yan et al. (2026) uses gene pathway prior knowledge as a semantic alignment anchor, maps pathology image patch features and gene expression features into a shared representation space, and progressively achieves coarse-to-fine alignment through self-attention and cross-attention modules. At the same time, this method adopts data augmentation strategies to adapt model training to small-scale tumor cohorts. For uncertainty quantification, general strategies such as integrating models trained with multiple random seeds, or adding slight noise perturbations to inputs and observing the output changes, can be adopted. Zheng et al. (2023) averaged the prediction results of multiple CT image cubes from the same patient to reduce the impact of local image heterogeneity on predictions, thereby improving the robustness of model outputs. These methods can provide a reference for predictive confidence in clinical decision-making, but attention should be paid to their computational cost and calibration requirements during application.

##### 8.1.2.6 Cross-architecture strategies

In addition to the architecture-specific recommendations above, the following strategies are applicable to most architectures. Samaras et al. (2026) developed MedScanGAN, a conditional generative adversarial network that creates high-fidelity synthetic PET and CT images to improve model training. Using these images for data augmentation raised the NSCLC classification accuracy of YOLOv8 from 92.30% to 94.14%. For privacy-preserving multi-center collaboration, Liu et al. (2024) built a federated learning model for radiotherapy response prediction using CT images from 245 NSCLC patients across four centers, with its AUC (0.689–0.725) proved comparable to that of a centralized deep learning model (0.695–0.718). Advancing this line of work, Huang et al. (2025) further introduced the FedCPI federated learning framework, which combines large and small models in a federated setting, and achieved an AUC of 0.9255 in external validation on 926 early-stage NSCLC patients from four centers. To safeguard reproducibility and clinical applicability, all prognostic models should follow the TRIPOD and TRIPOD + AI reporting guidelines.

### 8.2 Future research directions

To overcome the present technical obstacles, further investigations can be performed in several ways as follows.

1. Foundation model: based on current practical experience in natural language processing, the utilization of data like unlabeled medical images, pathological images, and genomic data to finish the pre-training of large-scale foundation models via self-supervised learning represents a promising direction in future. These models can contribute to feature initialization and adapt to a lot of downstream tasks. And the prognosis model performance in small-sample size scenarios may be enhanced through fine-tuning (Moor et al., 2023). Foundation models pre-trained on large-scale unlabeled data via self-supervised learning acquire generalizable representations that can be fine-tuned for downstream tasks, offering a new pathway to overcome the bottlenecks of limited sample sizes and scarce annotations in NSCLC prognosis prediction. BiomedCLIP, pre-trained in a domain-adaptive manner on the PMC-15M dataset, has achieved highly competitive results in image retrieval, classification, and visual question answering, and surpassed the radiology-specific model BioViL in the RSNA pneumonia detection task (Zhang S. et al., 2025). This model can serve as a feature extractor for CT, PET/CT, and pathological images, and holds promise for integration into existing multimodal prognostic frameworks; however, its specific efficacy in NSCLC prognosis remains to be validated. MedCLIP employs decoupled multimodal contrastive learning on image-text pairs, replacing the InfoNCE loss with a medical-knowledge-based semantic matching loss, effectively reducing false-negative interference and surpassing methods using approximately 200,000 data samples with only about 20,000 pre-training samples (Wang Z. et al., 2022). This paradigm accommodates imperfectly paired data across modalities, suggesting potential value in integrating clinically time-mismatched and multi-center non-standardized NSCLC data, though it has not yet been directly validated on NSCLC prognosis. CONCH, pre-trained on 1.17 million pathology image-text pairs, has achieved state-of-the-art performance across 14 tasks including histological image classification, segmentation, and image-text retrieval (Lu et al., 2024). A systematic evaluation of 19 pathology foundation models by Neidlinger et al. revealed that CONCH outperformed all vision-only models in overall performance, followed by Virchow2, and that their ensemble surpassed the single best model in 55% of tasks, also suggesting that data diversity may contribute more than data scale to performance (Neidlinger et al., 2025). This benchmark study covers multiple cancer types including lung cancer, providing a basis for model selection in NSCLC prognosis prediction. BEPH, pre-trained with masked image modeling on 11 million unlabeled pathology images, can be fine-tuned for tasks such as cancer diagnosis and subtype-specific survival prediction (Yang et al., 2025). Its pre-training-fine-tuning paradigm offers a technical pathway for prognostic modeling of NSCLC molecular subtypes with scarce data. MUSK, integrating 50 million WSI and 1 billion pathology image-text pairs through a two-stage pre-training process for image-text alignment, has demonstrated excellent performance on 23 benchmark evaluations (Xiang et al., 2025). MUSK has shown robust performance in predicting immunotherapy response in lung cancer and in refined risk stratification of lung cancer survival within the same pathological stage, providing strong support for the direct application of vision-language foundation models to NSCLC prognosis prediction. Large language models also demonstrate potential. Chang Y.-C. et al. (2025) fine-tuned LLaMA 3 on approximately 20,000 pathology reports (including 4,600 lung cancer patients), achieving a 70% F1 score for 3-year survival prediction, with external validation confirming cross-hospital robustness, addressing to some extent the challenge of efficiently extracting prognosis-relevant features from unstructured text. The generalizable representations acquired by foundation models hold potential for advancing NSCLC prognosis prediction through improved representation learning, facilitated multimodal fusion, and enhanced generalization. However, prominent challenges remain: *post hoc* attention heatmaps are not guaranteed to be consistent with the decision-making process; cross-center heterogeneity and the lack of large-scale prospective validation are more pronounced, with the advantage of optimal models significantly diminishing in low-data scenarios and low-prevalence tasks (Neidlinger et al., 2025); and the massive parameter sizes limit clinical accessibility. Future work should design refined adaptation strategies specific to NSCLC prognosis tasks, and conduct multi-center external validation and rigorous interpretability assessments following the TRIPOD + AI guidelines, so as to translate these models into trustworthy clinical tools.
2. Causal inference: most models have not addressed the fundamental causal linkages and still concentrate on learning correlations within data. The accurate identification of treatment target-related biological pathways affecting prognosis can be achieved via combining causal inference approaches with deep learning techniques. This will be conducive to the construction of predictive models more adaptive to clinical changes (Pearl, 2019).
3. Integrating biomedical prior knowledge: the incorporation of prior information from biomedical fields into network structure design can not only enhance the model interpretability but effectively reduce the dependence on annotated data.
4. Federated learning: It is possible to accomplish multi-institutional collaborative modeling without exchanging raw patient data, ensuring the security and privacy. This method offers a feasible means to develop large-scale multi-center prognosis models (Huang et al., 2025).
5. Dynamic prognosis and longitudinal data integration modeling: At present, the majority of models merely utilize the baseline data prior to treatment for static prediction, making it challenging to account for the dynamic changes in treatment response and illness progression. Tumors are spatiotemporal dynamic, and a great deal of prognostic data can be derived from the temporal changes in imaging and molecular markers after treatment. Dynamic risk classification and real-time patient prognosis evaluation can be realized by integrating various follow-up data via RNNs or Transformer-based temporal models. This conforms better to standard clinical diagnostic and therapeutic protocols (Lu et al., 2023).
6. Generative model-assisted data augmentation: generative models like GANs and variational autoencoders can be applied to generate synthetic data and impute missing information, which is conducive to the resolution of insufficient sample size problem (Karras et al., 2021; Koshino et al., 2021).

In the long run, prognostic models will gradually support the personalized treatment recommendation. By simulating how individual patients are likely to respond to different treatment strategies, these models can assist individualized decision-making, thereby promoting the implementation of precision medicine (Luo et al., 2026).

### 8.3 Bottlenecks and solutions for external validation

#### 8.3.1 Practical bottlenecks and lack of standardization in multi-center validation frameworks

Multi-center validation is a core step in evaluating the generalizability of deep learning prognostic models, but several bottlenecks currently exist in this field. First, a large number of models are still trained on single-center data and then validated on independent external cohorts, where the sample size and case composition of the validation cohort often differ significantly from those of the training set. Although the multi-modal deep learning model developed by Christie et al. (2025), which integrates PET/CT thoracic imaging with clinical and pathological information, employed an external validation cohort and could still significantly stratify patients into three risk subgroups in external validation, its AUC dropped from 0.78 on the test set to 0.66 on the external validation set. This performance degradation highlights that a single external validation is still insufficient to fully reflect the model’s actual performance in cross-center real-world settings. Hui (2025) constructed a DeepSurv model using multi-center resources, and the external validation C-index remained at 0.70. This relatively robust generalizability suggests that joint training with pooled multi-center raw data, despite regulatory barriers to data sharing, is indeed an effective path to improving generalization robustness.

A more fundamental issue is that differences in imaging acquisition protocols, pathological staining schemes, gene sequencing platforms, and analytical workflows across institutions introduce substantial technical batch effects unrelated to biological signals. Current traditional standardization methods, such as resampling to isotropic voxels, Z-score normalization, and ComBat batch correction, were originally designed for low-dimensional omics data, and their effectiveness in high-dimensional, nonlinear deep learning feature spaces has not been systematically verified. In the future, it is necessary to develop joint contrastive learning frameworks at the image data level to achieve cross-center domain-invariant feature extraction, and to introduce causal inference-based domain generalization methods at the feature level, so as to fundamentally reduce the interference of batch effects.

#### 8.3.2 Absence of prospective validation studies and the gap in the clinical evidence cascade

For deep learning prognostic models to achieve clinical translation, they must undergo a complete chain of evidence from retrospective development to prospective validation. However, prospective validation studies are severely lacking in the current field of deep learning-based prognostic prediction for NSCLC. While the number of clinical prediction models developed for NSCLC has grown rapidly, moving them into real-world clinical use remains a major challenge. The main bottleneck is the urgent need for external validation, combined with a shortage of truly prospective modeling. This gap in the evidence cascade leaves most models stuck at the stage of theoretical validation, unable to advance to actual deployment within clinical decision support systems. Even though the TRIPOD + AI guidelines have explicitly updated the term “validation” in the original TRIPOD statement to “evaluation” to reduce ambiguity and have strengthened requirements for complete reporting of machine learning models. Even so, prospective prognostic model studies that strictly follow these guidelines for prospective registration and reporting are extremely rare to date.

#### 8.3.3 Federated learning methods: solutions and limitations for data silos

Federated learning enables multiple centers to collaboratively build models without exchanging raw data, offering unique promise in protecting data privacy and improving cross-center model generalizability. In the field of NSCLC prognosis, however, the application of this technology remains in an early exploratory phase. For example, Liu Y. et al. (2025) proposed a robust federated learning model (RFed), based on data from 926 early-stage NSCLC surgical patients across four centers. The model achieved AUCs of 0.936, 0.861, 0.925, and 0.970 on each center’s test set, on the test set of each respective center, providing preliminary validation of federated learning’s feasibility for multicenter collaborative evaluation. Huang et al. (2025) further introduced the Federated cross-scale Common-Personal-Interactive learning framework (FedCPI), which incorporates feature decomposition and fusion between large and small models together with a federated adaptive communication strategy. On a four-center task, FedCPI attained a maximum AUC of 0.9255 and an accuracy of 0.8909, and its generalizability was preliminarily examined through cross-cancer-type validation.

In practice, federated learning still runs into serious difficulties. One major problem is that data across different centers tends to be highly non-independently and identically distributed. For example, the proportion of lung cancer subtypes and the distribution of TNM stages can vary considerably from one center to another. As a result, after federated averaging, the global model may actually perform worse than the individual local models. In addition, there is a significant gap between the standardized data format assumptions commonly adopted in current federated learning research and the process heterogeneity in real-world multi-center scenarios. The study by Liu et al. (2024) suggests that even within a federated learning framework, differences in feature space distributions across multiple centers may still lead to performance degradation when the model processes out-of-distribution data: when switching to external validation on datasets from different sources, the performance of the federated learning model is affected. Therefore, for federated learning to truly achieve the transition from “proof-of-concept” to “clinical deployment” in multi-center collaborative modeling, it is necessary to introduce communication efficiency optimization (e.g., parameter-selective synchronization in heterogeneous model federation) and model heterogeneity adaptation mechanisms (e.g., personalized federated learning) at the algorithm level, rather than simply relying on traditional federated averaging architectures.

#### 8.3.4 Real-world implementation barriers: from performance validation to multidimensional hurdles in clinical adoption

Even after deep learning prognostic models pass rigorous multicenter external validation, bringing them into real-world clinical practice still requires navigating several implementation barriers. The first is the narrow way model performance is typically validated. Most studies report only discrimination metrics like the C-index or AUC, while neglecting calibration—a measure of how closely predicted probabilities match actual observed risk. Calibration is essential for threshold-based clinical decisions, and risk models that have not been assessed for calibration can easily mislead treatment choices. The TRIPOD + AI guidelines now explicitly recommend reporting calibration curves, the Brier Score, or similar calibration metrics, yet most prognostic deep learning studies in NSCLC still do not follow this guidance. The second barrier is workflow integration: deploying these models demands clinical IT systems that support real-time image preprocessing, model inference, and results visualization, but interface standards between PACS and AI modules remain inconsistent across most healthcare institutions, resulting in inefficient data transfer. The third is a trust barrier—many clinicians have low trust in “black-box” decision-making, which is a core factor limiting adoption. Even when a model outperforms traditional methods on every quantitative metric, if its reasoning can’t be presented in a clinically interpretable way, it will struggle to find a place in routine clinical pathways. To overcome these barriers, future work should bring clinical-needs-oriented design thinking into the development stage, rigorously follow the TRIPOD + AI framework at the validation stage by reporting discrimination, calibration, and DCA in full, and turn to implementation science at the deployment stage to systematically evaluate how these models actually influence physician decision-making and patient outcomes within real clinical workflows.

#### 8.3.5 Potential solutions for improving model robustness, reproducibility, and clinical adoption

In multi-center settings, data heterogeneity poses the greatest risk to model robustness. Traditional approaches quickly lose their effectiveness in the high-dimensional feature spaces typical of deep learning. Federated learning, without sharing raw data, enhances cross-center generalization through collaborative training. Liu Y. et al. (2025) constructed the RFed model, achieving AUCs of 0.861–0.970 in four-center early-stage NSCLC progression prediction. Huang et al. (2025) further proposed the FedCPI framework, incorporating a large–small model synergistic mechanism, reaching an AUC of 0.9255 with cross-cancer validation. For non-IID data, Xu et al. (2025) proposed a heterogeneous federated learning model allowing different architectures at each center. Li et al. (2020) introduced FedProx, which constrains local–global model deviations via a proximal term. Multimodal integration and continual learning will further expand the robustness boundary of federated learning.

In terms of fusion strategies, decision-level (late) fusion exhibits stronger cross-center robustness than early and feature-level fusion. Zhao J. et al. (2025), in a multi-center study, showed that the decision-level fusion model achieved an external validation AUC of 0.812, outperforming single-modality and early fusion. Regarding interpretability, a novel framework (XplainLungSHAP) combining SHAP proposed by Costi et al. (2025) integrates SHAP attribution with attention mechanisms, achieving 91.49% accuracy in 1-year post-surgery survival prediction and significantly enhancing clinicians’ trust in model decisions. Interpretability is no longer merely a *post hoc* add-on but should become an integral part of model design.

The core of reproducibility lies in standardized reporting and comprehensive validation. Smiley et al. (2025) pointed out that although lung cancer AI studies account for the highest proportion (15.6%), there are notable deficiencies in sample size calculation, outlier handling, and performance heterogeneity reporting. Park et al. (2021) emphasized that calibration (not just discrimination) is crucial for clinical decision-making; models lacking calibration assessment may misguide treatment choices. Therefore, discrimination (C-index/AUC), calibration (Brier score/calibration curves), and DCA should together constitute a standard validation report.

Even with excellent model performance, clinical adoption still faces multiple barriers, including workflow integration and user trust. You et al. (2025) proposed a four-stage AI deployment framework: safety assessment, effectiveness validation, effectiveness evaluation and benchmark comparison, along with continuous monitoring, providing a pathway for large-scale deployment. Implementation science offers theoretical tools to bridge the “last mile” between AI innovation and clinical application. Fayaz-Bakhsh et al. (2026) noted that the average delay from AI development to real-world adoption may span several years, and implementation science is precisely the methodological foundation to fill this gap. Peek et al. (2024) identified workflow adaptability, system usability, and user trust as key drivers of adoption. Goodwin et al. (2025) recommended using the CFIR framework (covering five dimensions: intervention characteristics, outer and inner settings, individual characteristics, and implementation process) to guide model development and deployment, incorporating clinical workflow requirement analysis from the development stage, and enhancing physician decision confidence through human–computer interaction interfaces (e.g., parallel presentation of risk scores and attention heatmaps).

## 9 Conclusion

The research paradigm for NSCLC prognosis prediction has been deeply affected by deep learning technologies. Various approaches have boosted the prediction accuracy and elucidation of tumor biological behaviors, including CNNs for imaging processing, MIL for pathological images, GCNs for genomic/biological network modeling, as well as sophisticated multimodal fusion networks. In this paper, we reviewed the core technologies within this field: including architectural designs for different data modalities, multimodal fusion strategies from simple concatenation to deep interaction, along with multiple model interpretability approaches seeking to uncover the “black box.”

With the development of cutting-edge technologies like foundation models, causal inference, and dynamic modeling, as well as extensive multidisciplinary collaboration, deep learning is anticipated to become an essential decision-support tool for the accurate diagnosis and treatment of NSCLC in the future, although there still exist significant challenges in data standardization, model generalization, validation of interpretability, and clinical translation, etc. Prognostic assessment will be further driven toward precision, individualization, and interpretability by next-generation models emphasizing robust validation, causal modeling, dynamic evaluation, and knowledge integration. This will ultimately improve the survival and life quality of NSCLC patients.
